# Level pinning of anti-*PT*-symmetric circuits for efficient wireless power transfer

**DOI:** 10.1093/nsr/nwad172

**Published:** 2023-06-14

**Authors:** Zhiwei Guo, Fengqing Yang, Haiyan Zhang, Xian Wu, Qiong Wu, Kejia Zhu, Jun Jiang, Haitao Jiang, Yaping Yang, Yunhui Li, Hong Chen

**Affiliations:** MOE Key Laboratory of Advanced Micro-Structured Materials, School of Physics Sciences and Engineering, Tongji University, Shanghai200092, China; MOE Key Laboratory of Advanced Micro-Structured Materials, School of Physics Sciences and Engineering, Tongji University, Shanghai200092, China; MOE Key Laboratory of Advanced Micro-Structured Materials, School of Physics Sciences and Engineering, Tongji University, Shanghai200092, China; MOE Key Laboratory of Advanced Micro-Structured Materials, School of Physics Sciences and Engineering, Tongji University, Shanghai200092, China; MOE Key Laboratory of Advanced Micro-Structured Materials, School of Physics Sciences and Engineering, Tongji University, Shanghai200092, China; Department of Electrical Engineering, Tongji University, Shanghai201804, China; School of Automotive Studies, Tongji University, Shanghai210804, China; MOE Key Laboratory of Advanced Micro-Structured Materials, School of Physics Sciences and Engineering, Tongji University, Shanghai200092, China; MOE Key Laboratory of Advanced Micro-Structured Materials, School of Physics Sciences and Engineering, Tongji University, Shanghai200092, China; Department of Electrical Engineering, Tongji University, Shanghai201804, China; MOE Key Laboratory of Advanced Micro-Structured Materials, School of Physics Sciences and Engineering, Tongji University, Shanghai200092, China

**Keywords:** non-Hermitian system, wireless power transfer, anti-*PT*-symmetric circuits, synthetic dimension, metamaterials

## Abstract

Wireless power transfer (WPT) technology based on magnetic resonance (a basic physical phenomenon) can directly transfer energy from the source to the load without wires and other physical contacts, and has been successfully applied to implantable medical devices, electric vehicles, robotic arms and other fields. However, due to the frequency splitting of near-field coupling, the resonant WPT system has some unique limitations, such as poor transmission stability and low efficiency. Here, we propose anti-resonance with level pinning for high-performance WPT. By introducing the anti-resonance mode into the basic WPT platform, we uncover the competition between dissipative coupling and coherent coupling to achieve novel level pinning, and construct an effective anti-parity-time (anti-*PT*)-symmetric non-Hermitian system that is superior to previous *PT*-symmetric WPT schemes. On the one hand, the eigenvalue of the anti-*PT*-symmetric system at resonance frequency is always pure real in both strong and weak coupling regions, and can be used to overcome the transmission efficiency decrease caused by weak coupling, as brought about by, for example, a large size ratio of the transmitter to receiver, or a long transmission distance. On the other hand, due to the level pinning effect of the two kinds of coupling mechanisms, the working frequency of the system is guaranteed to be locked, so frequency tracking is not required when the position and size of the receiver change. Even if the system deviates from the matching condition, an efficient WPT can be realized, thereby demonstrating the robustness of the level pinning. The experimental results show that when the size ratio of the transmitter coil to the receiver coil is 4.29 (which is in the weak coupling region), the transfer efficiency of the anti-*PT*-symmetric system is nearly 4.3 (3.2) times higher than that of the *PT*-symmetric system when the matching conditions are satisfied (deviated). With the miniaturization and integration of devices in mind, a synthetic anti-*PT*-symmetric system is used to realize a robust WPT. Anti-*PT*-symmetric WPT technology based on the synthetic dimension not only provides a good research platform for the study of abundant non-Hermitian physics, but also provides a means of going beyond traditional near-field applications with resonance mechanisms, such as resonance imaging, wireless sensing and photonic routing.

## Teaser

Using anti-resonance mode instead of resonance mode, a synthetic third-order non-Hermitian circuit with anti-PT symmetry is constructed, and wherein the novel level pinning effect enables efficient wireless power transfer.

## INTRODUCTION

As a promising technology that utilizes electromagnetic waves to convey power from the source to the load directly without cables, wireless power transfer (WPT) opens up a novel way for people to utilize electric energy [1,2]. WPT, with the advantages of safety, reliability and lifespan, has sparked tremendous research interest in a wide range of electronic device applications, such as mobile phones [3], mobile manipulators [4], medically implanted devices [5] and electric vehicles [6]. However, traditional WPT is seriously limited by transmission distance. In 2007, Kurs *et al.* achieved midrange efficient energy transmission via strongly near-field-coupled magnetic resonances between two coils [7]. This pioneering work has aroused great interest, and recently, non-radiative magnetic resonance WPT has been extensively developed [8,9]. With the help of novel physical concepts, modern WPT technologies have been significantly developed [10], including non-Hermitian physics [11–19], topological photonics [20–22], quantum optics [23–26], novel localized states [27–29] and metamaterials [30–32]. The typical magnetic resonance WPT scheme based on near-field coupling corresponds to a configuration of ‘RTC*-*NFC-RRC’, where RTC, NFC and RRC denote the resonance transmitter coil, near-field coupling and resonance receiver coil, respectively. However, the working frequency in the conventional WPT system is usually very sensitive to operational conditions, thus the operating frequency needs to be tuned with the position and size of the receiver because strong NFC will always lead to the working frequency splitting [33]. Therefore, a long-standing problem has been the laborious split-frequency tracking that should be used for the system to be kept on resonance with a varying operational condition. To overcome the issue of frequency tracking, optimization strategies are proposed, for example, using the controlling circuit and feedback circuit to track the stable working frequency [34] or utilizing non-linearities [11,13,14]. Notably, in 2017, Assawaworrarit *et al*. experimentally revealed robust WPT using a non-linear parity-time (*PT*)-symmetric circuit (which incorporates a non-linear gain saturation element) to track the real-time working frequency, which offers a novel way to explore stable WPT with non-Hermitian physics [11]. It should be emphasized that although this scheme can lock different working frequencies when the position/size of the receiver changes, and exhibits excellent performance in the strong coupling region, efficient energy transfer in the weak coupling region is difficult. From the perspective of basic physical principles, the strong coupling region in the *PT-*symmetric system is limited by the exceptional point (EP), indicating *PT*-symmetry phase transition. Once the size ratio of transmitter to receiver increases or the transmission distance increases, causing a non-Hermitian system to move from a strong coupling region through the EP to a weak coupling region, there will be imaginary parts in the eigenvalues, resulting in a reduction of transfer efficiency in the WPT system [19]. Especially in the scenarios of mid-long-range consumer electronics [12,31] and medical implant devices with mismatched sizes [5,35], a large size ratio of the transmitter to receiver, or a long transmission distance energy transfer of technical requirements cannot be achieved through non-linearities and feedback circuits due to the basic physical limitations in *PT*-symmetric systems [11,34]. Moreover, from the perspective of practical applications, it is crucial to note that the WPT systems with non-linear circuit elements require technical input signals with very high power, and they are generally difficult to use in many high-power application scenarios. Therefore, a possible linear physical system for efficient and stable WPT urgently needs to be investigated.

In this work, we conduct a systematical study involving both theory and experiments on a new magnetic resonance WPT scheme with an anti-resonance transmitter beyond the conventional design. In contrast to the typical scheme ‘RTC-NFC-RRC’, the key configuration here, based on near-field coupling, corresponds to ‘ATC-NFC-RRC’, where ATC represents an anti-resonance transmitter coil. In this effective non-Hermitian WPT system composed of ATC and RRC, a third-order anti-*PT*-symmetry with novel level-pinning effect is established. The novel level pinning (the working frequency does not vary with the coupling strength) is enabled by the competition between the level attraction of the dissipative coupling mechanism (i.e. imaginary coupling $i\gamma $) and the level repulsion of the coherent coupling mechanism (i.e. real coupling $\kappa $), respectively [36,37]. Dissipative coupling and coherent coupling correspond to the resistive coupling [38] and capacitive (inductive) coupling [39] in the inductance, capacitance and resistance (LRC) circuit, respectively. The advantages of the near-field WPT scheme based on the ‘ATC-NFC-RRC’ configuration are mainly reflected in the following aspects. On the one hand, although there is anti-*PT*-symmetry and anti-*PT*-symmetry breaking phases identified by EPs in anti-*PT*-symmetric systems, the eigenvalue of the level pinning position is always pure real, indicating the stable transfer mode of the WPT system in both strong and weak coupling regions, which can be used for efficient energy transfer [11,19]. Therefore, anti-*PT*-symmetric systems can overcome transmission efficiency decrease due to weak coupling in *PT*-symmetric systems. On the other hand, the level-pinning effect achieved by competition between two coupling mechanisms ensures that the operating frequency of the system is locked, thus frequency tracking is not required when the position and size of the receiver change. Even if the system deviates from the perfect matching condition, the level-pinning effect in anti-*PT*-symmetric WPT systems still supports a mode with slight dissipation at the resonance frequency, thus its efficient transfer efficiency is more robust than the previous WPT schemes with *PT*-symmetry. Inspired by novel synthetic-dimensional physics, here we demonstrate a reconfigurable third-order non-Hermitian system with anti-*PT*-symmetry based on the meta-coil resonator (MCR). The synthetic third-order non-Hermitian system with anti-*PT*-symmetry in this work provides a way to realize efficient WPT. The related results can also be directly extended to other near-field applications, such as wireless sensing and communications.

## RESULTS

### Theoretical model

**Figure 1.**
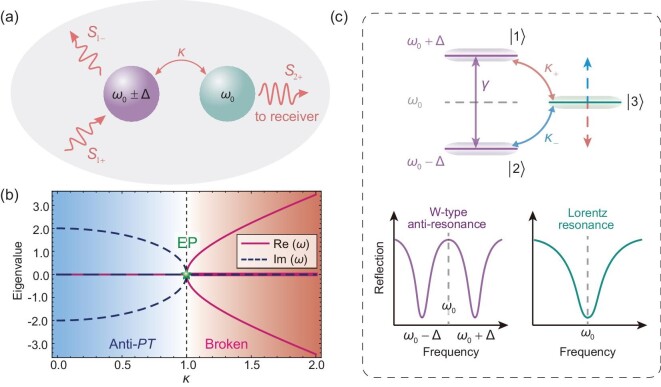
Schematic of the effective third-order anti-*PT*-symmetric system. (a) General physical model of the non-Hermitian system consisting of three coupled resonant modes, whose resonant frequencies correspond to ${\omega }_0 + {\mathrm{\Delta }}$, ${\omega }_0 - {\mathrm{\Delta }}$ and ${\omega }_0$, respectively. ${\mathrm{\Delta }}$ denotes the detuning factor. The coupling coefficient between the resonant mode ${\omega }_0$ and the detuning modes ${\omega }_0 \pm {\mathrm{\Delta }}$ is $\kappa $. The input, reflected and output signals are represented by ${S}_{1 + }$, ${S}_{1 - }$ and ${S}_{2 + }$ respectively. (b) Evolution of the real parts (solid line) and imaginary parts (dashed line) of the eigenfrequencies in the anti-*PT*-symmetric system. The third-order EP is marked by the star. (c) Three-level scheme of (a). The destructive interference between the detuning modes means that the position of the resonant mode ${\omega }_0$ is always maintained. The lower row gives the reflection spectra of two types of modes.

As a starting point, consider a special non-Hermitian system, in which an anti-resonance structure contains two detuned modes (${\omega }_ + = {\omega }_0 + \Delta $ and ${\omega }_ - = {\omega }_0 - \Delta $) that are coupled with the third resonance mode (${\omega }_0$, which is depicted in Fig. 1(a). The coupling strength of the detuned modes and the resonance mode is κ. $\gamma $ and $\Delta $ denote the dissipative coupling and detuning factor of the resonance frequency in the anti-resonance structure. As an open physical system, the dynamic equation of this system in Fig. 1(a) can be written as [11,19]:


(1) \begin{eqnarray*}
&& {\frac{da_{+}}{dt} = [ - i({\omega }_0 + \Delta ) - {\gamma }_ + - {\Gamma }_ + ]{a}_ + + \gamma {a}_ - + i{\kappa }_ + {a}_0 + \sqrt {2{\gamma }_ + } {s}_{ + {T}_1}} \\
&& {\frac{da_{-}}{dt} = [ - i({\omega }_0 - \Delta ) - {\gamma }_ - - {\Gamma }_ - ]{a}_ - + \gamma {a}_ + - i{\kappa }_ - {a}_0 + \sqrt {2{\gamma }_ - } {s}_{ - {T}_1}} \\
&& {\frac{da_{0}}{dt} = [ - i{\omega }_0 - {\gamma }_0 - {\Gamma }_0]{a}_0 + i{\kappa }_ + {a}_ + - i{\kappa }_ - {a}_ - },
\end{eqnarray*}

where ${\gamma }_j$ and ${\Gamma }_j$ ($j = + $,$- $,$0$) denote the dissipative loss and radiative loss of the harmonic modes ${a}_j = {A}_j{e}^{ - i\omega t}$, respectively. ${\kappa }_ \pm $ is the near-field coupling coefficient between the anti-resonance and resonance structures. ${s}_{ + {T}_1} = {S}_{ + {T}_1}{e}^{ - i\omega t}$ and ${s}_{ - {T}_1} = {S}_{ -{T}_1}{e}^{ - i\omega t}$ represent the external incoming waves to the detuned modes of the anti-resonance structure. Considering the zero reflected waves ${S}_{k{R}_1} = - {S}_{k{T}_1} + \sqrt {2{\gamma }_k} {a}_k = 0\,\,$$(k = + , - )$, the dynamics equation of the system is [40]:


(2) \begin{eqnarray*}
{\boldsymbol{H}}\left( {\begin{array}{@{}*{1}{c}@{}} {{a}_ + }\\ {{a}_0}\\ {{a}_ - } \end{array}} \right) = \omega \left( {\begin{array}{@{}*{1}{c}@{}} {{a}_ + }\\ {{a}_0}\\ {{a}_ - } \end{array}} \right).
\end{eqnarray*}

The Hamiltonian of the non-Hermitian system with anti-resonance structure can be written as follows [11,40]:


(3) \begin{eqnarray*}{\boldsymbol{H}} = \left( {\begin{array}{@{}*{3}{c}@{}} {{\omega }_0 + \Delta + i{\gamma }_ + - i{\Gamma }_ + }&{ - {\kappa }_ + }&{i\gamma }\\ { - {\kappa }_ + }&\quad {{\omega }_0 - i{\gamma }_0 - i{\Gamma }_0}&{{\kappa }_ - }\\ {i\gamma }&{{\kappa }_ - }&\quad {{\omega }_0 - \Delta + i{\gamma }_ - - i{\Gamma }_ - } \end{array}} \right).
\end{eqnarray*}

To simplify the system, considering the same dissipative loss ${\gamma }_ + = {\gamma }_ - = {\gamma }_0/2 = \gamma $, same near-field coupling coefficient ${\kappa }_ + = {\kappa }_ - = \kappa $, and ignoring the radiative loss ${\Gamma }_ + = {\Gamma }_ - = {\Gamma }_0 = 0$ for the harmonic modes, the Hamiltonian can be simplified as:


(4) \begin{eqnarray*}
{\boldsymbol{H}} = \left( {\begin{array}{@{}*{3}{c}@{}} {{\omega }_0 + \Delta + i\gamma }&{ - \kappa }&{i\gamma }\\ { - \kappa }&{{\omega }_0 - 2i\gamma }&\kappa \\ {i\gamma }&\kappa &{{\omega }_0 - \Delta + i\gamma } \end{array}} \right).
\end{eqnarray*}

From Equation (4), the anti-*PT*-symmetry of the third-order non-Hermitian system about the center ${\omega }_0$ is verified [41–46], i.e.:


(5) \begin{eqnarray*}
(PT){\boldsymbol{H}}{(PT)}^{ - 1} = P{{\boldsymbol{H}}}^*P = - {\boldsymbol{H}}{\boldsymbol{.}}
\end{eqnarray*}

It is precisely due to the real and imaginary coupling coefficients in the off-diagonal terms that the Hamiltonian of the system satisfies the anti-*PT*-symmetry condition, which makes the pure real eigenfrequency available for efficient energy transfer. According to Equation (4), the eigenvalues of the anti-*PT*-symmetric non-Hermitian system are found to be:


(6) \begin{eqnarray*}
{\omega }_1 &=& - \frac{{{2}^{1/3}A}}{{3{{(iB + \sqrt {4{A}^3 - {B}^2} )}}^{1/3}}} \\
&& +\, \frac{{(iB + \sqrt {4{A}^3 - {B}^2} )}}{{3 \times {2}^{1/3}}} + {\omega }_0,
\end{eqnarray*}

(7) \begin{eqnarray*}
{\omega }_2 &=& - \frac{{(1 + \sqrt 3 i)A}}{{3 \times {2}^{2/3}{{(iB + \sqrt {4{A}^3 - {B}^2} )}}^{1/3}}} \\&& +\, \frac{{(1 - \sqrt 3 i)(iB + \sqrt {4{A}^3 - {B}^2} )}}{{6 \times {2}^{1/3}}} +\, {\omega }_0,\\
\end{eqnarray*}

(8) \begin{eqnarray*}
{\omega }_3 &=& - \frac{{(1 - \sqrt 3 i)A}}{{3 \times {2}^{2/3}{{(iB + \sqrt {4{A}^3 - {B}^2} )}}^{1/3}}} \\ && +\, \frac{{(1 + \sqrt 3 i)(iB + \sqrt {4{A}^3 - {B}^2} )}}{{6 \times {2}^{1/3}}} +\, {\omega }_0,\\
\end{eqnarray*}

where $A = 3(4{\gamma }^2 - {\Delta }^2 - 2{\kappa }^2)$ and $B = 54\gamma ({\Delta }^2 - 2{\kappa }^2)$. The level pinning of the anti-*PT*-symmetric non-Hermitian system can be analytically demonstrated (more details about the level pinning of locking at the resonant frequency ${\mathop{\rm Re}\nolimits} ({\omega }_1) = {\omega }_0$ due to the competing couplings are introduced in the Supplementary Data). Although the level pinning of the working frequency at the resonant frequency always occurs due to the competing couplings, the pure real eigenvalues in general non-Hermitian systems cannot be guaranteed. When the dissipative coupling is not zero ($\gamma \ne 0$), from Equation (6) we can determine that the precondition for a real eigenfrequency of ${\boldsymbol{H}}$ at ${\omega }_1 = {\omega }_0$ is $\Delta = \sqrt 2 \kappa $. Therefore, the coexistence of two different types of coupling in the system is crucial. On the one hand, the competitive cancellation between them leads to level pinning, which ensures the working frequency locking. On the other hand, from the Hamiltonian given in Equation (4), the real and imaginary coupling coefficients are the key components for constructing the anti-*PT*-symmetric WPT system, which ensures efficient energy transfer based on the real eigenvalues at the working frequency $\omega = {{\mathrm{\omega }}}_0$. Without losing any generality, setting $\gamma = 1$, the evolution of the real parts and imaginary parts of the eigenfrequencies as a function of $\kappa $ in the hybrid system composed of anti-resonance and resonance modes can be obtained, as shown in Fig. 1(b). The third-order EP used to separate anti-*PT*-symmetric phase and broken phase is represented by the star. Recently, anti-*PT*-symmetric physics, emerging in non-Hermitian systems with unbalanced distributions between gain and loss, has drawn massive attention. So far, anti-*PT*-symmetric systems have been proposed, including cavity-magnonic coupling [36,37,47], the spinning resonator [48] and the fiber [49]. Moreover, the circuit-based system with lumped elements provides a flexible platform to construct the *PT*-symmetric [50–53] and anti-*PT*-symmetric [38] systems. In particular, anti-*PT-*symmetry has been extended to realize chiral polarizers [54] and topological structures [55,56]. However, the anti-*PT*-symmetric system based on the anti-resonance structure remains elusive. A three-level scheme of the anti-*PT*-symmetric system with an anti-resonance structure is shown in Fig. 1(c). It can be seen that the destructive interference between the detuning modes of the anti-resonance structure on the resonance mode ensures that the position of the resonant mode ${\omega }_0$ is always maintained. This locked eigenstate can be used to realize robust WPT.

**Figure 2.**
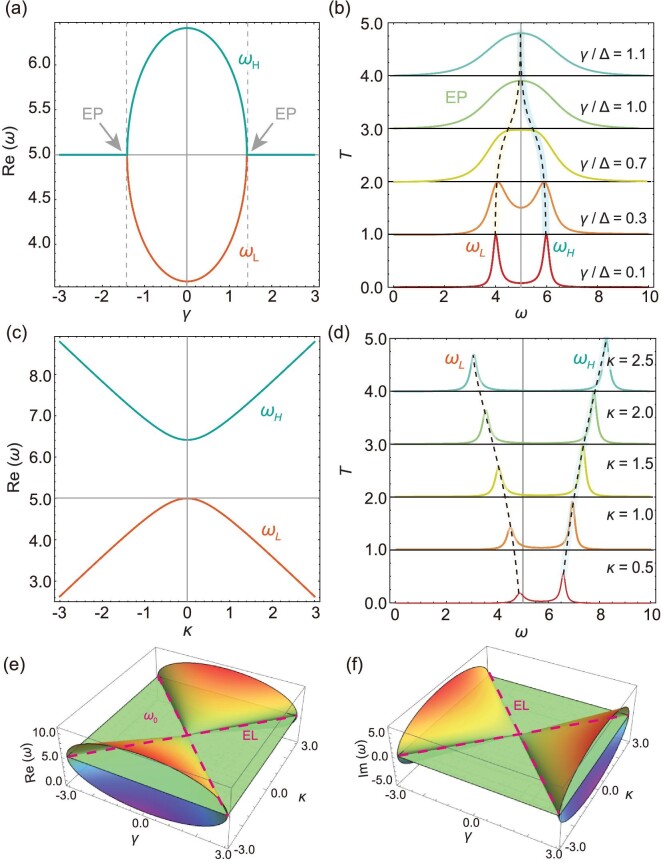
Level pinning of the anti-*PT*-symmetric non-Hermitian circuit with anti-resonance mode. (a and b) Level attraction in the anti-resonance mode with dissipative coupling. (a) Evolution of the real parts of the eigenfrequencies in the anti-resonance system. (b) Calculated transmission spectra of the anti-resonance mode for the different normalized dissipative coupling ${\mathrm{\gamma }}/{\mathrm{\Delta }}$. The transmission peaks are connected by black dashed lines to show the level attraction effect. (c and d) Level repulsion between the resonance modes of the anti-resonance and resonance structures. (c) Evolution of the real parts of the eigenfrequencies in the system with two resonance modes ${\omega }_0 + {\mathrm{\Delta }}$ and ${\omega }_0$. (d) Calculated transmission spectra for the different coherent coupling ${\mathrm{\kappa }}$ between resonance mode ${\omega }_0 + {\mathrm{\Delta }}$ and resonance mode ${\omega }_0$. The transmission peaks are connected by black dashed lines to show the level repulsion effect. (e and f) Level pinning in the system composed of anti-resonance and resonance modes. Evolution of the real parts (e) and imaginary parts (f) of the eigenfrequencies as a function of dissipative coupling ($\gamma )$ and coherent coupling ($\kappa $) in the hybrid system composed of anti-resonance and resonance modes. The exceptional lines (ELs) connected by EPs are marked by the dashed lines. The plane represents $\omega \ = \ {\omega }_0$.

The pinning level $\omega = {\omega }_0$ in the system is controlled by the level attraction between the resonance modes (${\omega }_ + $ and ${\omega }_ - $) in the anti-resonance structure, and the upward and downward repulsion of the resonance frequency ${\omega }_0$ comes from ${\omega }_ + $ and ${\omega }_ - $, respectively. In addition, it should be pointed out that the *P*-symmetry here is about the frequency space rather than the usual position space [12]. Therefore, the novel level pinning of the third-order anti-*PT*-symmetric system is enabled by the competition between the level attraction of dissipative coupling and the level repulsion of coherent coupling. Based on the physical picture of the anti-*PT*-symmetric system established above, we next quantitatively introduce the novel level pinning caused by competition between coherent and incoherent effects. For the single ‘W-type’ anti-resonance structure, the evolution of the real parts of the eigenfrequencies and analytical outgoing transmittance spectra for different dissipative couplings are shown in Fig. 2(a), respectively. It can be clearly seen that with the increase of the normalized dissipative coupling $\gamma /\Delta $, two split transmission peaks gradually approach each other and finally coalesce at the EP ($\gamma /\Delta = 1$), which indicates the level attraction of dissipative coupling [36,37,47]. In contrast, the level repulsion due to the coherent coupling between the resonance modes in the anti-resonance and resonance structures can also be uncovered directly from the transmittance spectra. Taking the mode ${\omega }_ + = {\omega }_0 + \Delta $ in the anti-resonance structure as an example, the evolution of the real parts of the eigenfrequencies and the corresponding transmission spectra of the coupled resonance modes in the anti-resonance structure (${\omega }_ + $) and resonance structure (${\omega }_0$) are shown in Fig. 2(c) and (d), respectively. With the increase of coherent coupling, the splitting modes gradually move away, which exhibits the conventional level repulsion of coherent coupling [7,11]. Importantly, the competition between dissipative coupling and coherent coupling of the third-order anti-*PT*-symmetric system in Fig. 1 leads to novel level pinning. According to Equations (6–8), the evolution of the real parts and imaginary parts of the eigenfrequencies as a function of dissipative coupling and coherent coupling can be obtained in the hybrid system composed of anti-resonance and resonance modes, as shown in Fig. 2(e) and (f), respectively. On the Riemannian surface, composed of three parameters ($\omega $, $\gamma $ and $\kappa $), we can clearly see a stable plane $\omega = {\omega }_0$ independent of the coupling coefficients $\gamma $ and $\kappa $, which comes from the competition between dissipative coupling and coherent coupling in the system composed of anti-resonance and resonance modes. The exceptional lines (ELs) connected by EPs with $\kappa = \gamma $ are marked by the dashed lines. With the aid of novel level pinning and a locked real eigenvalue in the anti-*PT* symmetric system, stable and efficient WPT can be achieved.

### Experimental realization

#### Synthetic anti-*PT*-symmetric non-Hermitian WPT system based on the anti-resonance circuit

**Figure 3.**
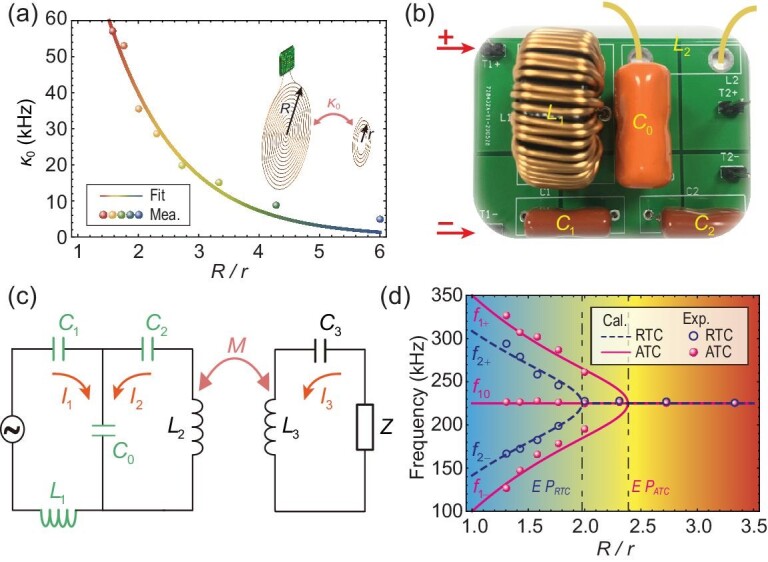
Synthetic third-order anti-*PT*-symmetric WPT system. (a) The exponential decreases of the coupling strength with the radius ratio of synthetic ATC and RRC. The radius of the synthetic ATC is fixed at $R = 15$ cm. The measured data and the fitted line (${{\mathrm{\kappa }}}_0 = 159.55{{\mathrm{e}}}^{ - 0.72R/r}$ kHz) are shown by the dots and the solid line, respectively. The structure and related parameters are shown in the inset. (b) Photograph of the experimental lumped electronic components for the synthetic ATC. The signal is input from the ‘+’ and ‘−’ on the left. The distributed inductance ${L}_2$ is provided by the coil. Here, ${L}_1 = {L}_2 = L$ and ${C}_1 = C{C}_0/( {{C}_0 - C} ).$ (c) The effective circuit model of the synthetic third-order anti-*PT*-symmetric WPT system, where the ATC (left circuit) and RRC (right circuit) are coupled with the help of mutual inductance *M* between ${L}_2$ and ${L}_3$. (d) Evolution of the calculated (measured) real parts of eigenfrequencies in the synthetic third-order anti-*PT*-symmetric non-Hermitian system as a function of the radius ratio of synthetic ATC and RRC, which is marked by the solid line (solid spheres). For comparison, the calculated (measured) phase diagram of conventional WPT with two coils (second-order *PT*-symmetric system) is also given by the dashed line (hollow circles). The EPs of the conventional WPT system with RTC and the novel ATC are marked by the $E{P}_{RTC}$ and $E{P}_{ATC}$, respectively.

In addition to stability, technologies also require high efficiency, integration, miniaturization and multi-loads, which are problems to be urgently solved in practical application. Inspired by novel synthetic-dimensional physics [57–61], here we demonstrate a reconfigurable third-order non-Hermitian system with anti-*PT*-symmetry based on a planar artificial MCR with unusual electromagnetic properties [28,60,61]. The schematic of the synthetic anti-*PT*-symmetric WPT system is shown in the inset of Fig. 3(a). The radius of the synthetic ATC is fixed at $R = $15 cm. Under the near-field coupling mechanism, the coupling strength between the MCR-based ATC and the RRC decreases exponentially with the radius ratio of synthetic ATC and RRC as ${\kappa }_0 = 159.55{e}^{ - 0.72R/r}$ kHz (details are provided in the Supplementary Data), which is shown in Fig. 3(a). A photograph of the experimental lumped electronic components for the synthetic ATC is shown in Fig. 3(b). We can see that the signal is input from the ‘+’ and ‘−’ on the left of the circuit board. Here, ${L}_2$ is the distributed inductance, which is provided by the wire structure, while ${L}_1$ and ${C}_i$ ($i = 0,1,2$) are lumped inductance and capacitor, respectively. Figure 3(c) shows the corresponding effective circuit model of the synthetic third-order anti-*PT*-symmetric WPT system, where the ATC (left circuit) and RRC (right circuit) are coupled with the help of mutual inductance *M* between the distributed inductance ${L}_2$ and ${L}_3$. Considering ${L}_1 = {L}_2 = {L}_3 = L$, ${C}_2 = {C}_1$ and ${C}_3 = C = {C}_0{C}_1/({C}_0 + {C}_1)$, we can get the dynamic equation of the synthetic anti-*PT*-symmetric system as (more details about the dynamic equation that derived from Kirchhoff's equations are introduced in the Supplementary Data):


(9) \begin{eqnarray*}
\left( {\begin{array}{@{}*{3}{c}@{}} {\displaystyle\frac{1}{{\sqrt {LC} }} + \displaystyle\frac{1}{{2\omega {C}_0L}} + \displaystyle\frac{{{\gamma }_0}}{2}i}&\quad {\displaystyle\frac{{ - M\omega }}{{2\sqrt 2 L}}}&{\displaystyle\frac{{{\gamma }_0}}{2}i}\\ {\displaystyle\frac{{ - M\omega }}{{2\sqrt 2 L}}}&\quad {\displaystyle\frac{1}{{\sqrt {LC} }} - i{\gamma }_0}&{\displaystyle\frac{{M\omega }}{{2\sqrt 2 L}}}\\ {\displaystyle\frac{{{\gamma }_0}}{2}i}&{\displaystyle\frac{{M\omega }}{{2\sqrt 2 L}}}&{\displaystyle\frac{1}{{\sqrt {LC} }} - \displaystyle\frac{1}{{2\omega {C}_0L}} + \displaystyle\frac{{{\gamma }_0}}{2}i} \end{array}} \right)\left( {\begin{array}{@{}*{1}{c}@{}} {{a}_ + }\\ {{a}_0}\\ {{a}_ - } \end{array}} \right) = \omega \left( {\begin{array}{@{}*{1}{c}@{}} {{a}_ + }\\ {{a}_0}\\ {{a}_ - } \end{array}} \right),
\end{eqnarray*}

where $M = \xi L$ denotes the mutual inductance between the synthetic ATC and RRC. $\xi = C/{C}_0$ is the coupling factor for different loads. The effective loss is ${\gamma }_0 = - Z/2L$. We assume ${\omega }_0 = 1/\sqrt {LC} $, $\gamma = {\gamma }_0/2$, $\Delta = 1/2\omega {C}_0L$, $\kappa = M\omega /2\sqrt 2 L$, and Equation (10) can be reduced as Equation (4), thus the non-Hermitian WPT system with ATC and RRC satisfies the third-order anti-*PT*-symmetry about the center ${\omega }_0$. In practice, we expect that WPT devices will be reliable for diverse loads of different sizes. Here, $L = 98\,\,\mu H$, $C = 5.1\,\,nF$ and $Z = 50\,\,\Omega $. With the change of radius ratio of synthetic ATC and RRC, the calculated and measured evolution of the real parts of eigenfrequencies in the synthetic third-order anti-*PT*-symmetric non-Hermitian system is given, shown by the pink solid line and solid spheres in Fig. 3(d), respectively. We can see that the eigenfrequency ${f}_0 = {\omega }_0/2\pi = 225$ kHz remains independent of the radius of RRC. In addition, the eigenfrequencies coalesce at an EP_ATC_ with *R/r* = 2.41 for the synthetic third-order anti-*PT*-symmetric non-Hermitian system. For comparison, the calculated (measured) phase diagram of conventional RTC under the same parameters is also given by the dashed line (hollow circles) in Fig. 3(d). For the RTC case, the eigenfrequencies coalesce at an EP_RTC_ with *R/r* = 1.98, while *R/r* deviating from this critical value will lead to the rapid decrease of transfer efficiency (more details about the relation between the real eigenfrequency and the transfer efficiency for the RTC case are shown in the Supplementary Data). From Fig. 3(d), it can be clearly seen that the radius ratio of the EP in the ATC system is larger than that of the RTC system under the same parameters.

#### Level pinning of the anti-*PT*-symmetric WPT system for robust WPT

The transfer efficiency of the anti-*PT*-symmetric WPT system with the ‘W-type’ anti-resonance mode and Lorentz resonance mode can be expressed as:


(10) \begin{eqnarray*}
\eta &=& {\left| {\frac{{{S}_{2 + }}}{{{S}_{1 + }}}} \right|}^2 \\
&=& {\left| {\frac{{2\sqrt 2 {\gamma }_0\Delta \kappa }}{{\left[ {{\gamma }_0 - i(\omega - {\omega }_0)} \right][{\Delta }^2 + 2{\kappa }^2 - (\omega - {\omega }_0)(i{\gamma }_0 + \omega - {\omega }_0)]}}} \right|}^2. \\
\end{eqnarray*}

**Figure 4.**
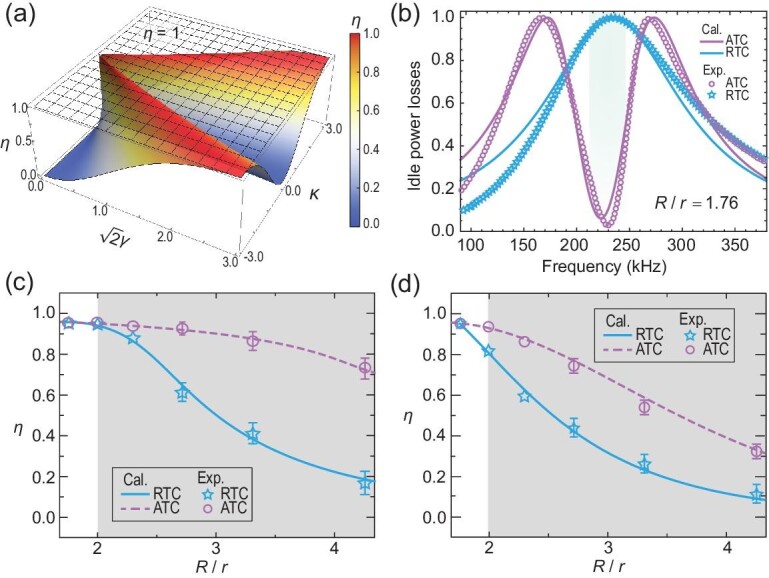
Efficient and stable WPT achieved by the level-pinning effect of an anti-*PT*-symmetric non-Hermitian system. (a) A comparison of transfer efficiency for the WPT system with RTC and ATC at the working frequency $\omega \ = \ {\omega }_0$, marked by the solid and mesh surfaces, respectively. (b) Idle power losses of the WPT systems with $R/r\ = \ 1.76$. The calculated (measured) results of the WPT systems with RTC and ATC are marked by the solid line (stars) and the dashed line (circles), respectively. Compared with the RTC scheme, the frequency range of the ATC scheme with significantly lower idle power loss is represented by the shaded area. (c) Transfer efficiency of the matched WPT system with RTC (ATC) as a function of $R/r$ at the tracked working frequencies (a fixed working frequency 225 kHz). The calculated and measured results of the WPT system with RTC (ATC) are shown by the solid line (dashed line) and stars (circles), respectively. The weak coupling region of the WPT system with RTC is denoted by shading. (d) Same as (c), but for the transfer efficiency versus $R/r$ in the general WPT system with RTC (ATC) at a fixed working frequency 198.3 kHz (225 kHz).

From Equation (10), the optimized transfer efficiency $\eta = 1$ can always be achieved at the fixed resonance frequency ($\omega = {\omega }_0$) when the matching condition $\Delta = \sqrt 2 \kappa $ is satisfied. In other words, under this matching condition, a stable and efficient WPT independent of the coupling parameters can be realized in the anti-*PT*-symmetric WPT system. Comparing Equations (6) and (10), it can be found that the stable power transfer state in the WPT system with anti-resonance mode corresponds to a real eigenvalue of the effective Hamiltonian for an anti-*PT*-symmetric non-Hermitian system. Moreover, in order to determine the transfer efficiency of the non-Hermitian WPT system with anti-resonance mode, Fig. 4(a) gives the comparison of transfer efficiency for the WPT system with RTC and ATC at a fixed working angular frequency $\omega = {\omega }_0$. The optimized transmission efficiency corresponds to a stable WPT system with ATC based on the level pinning, as shown by the black mesh surface. In contrast, the solid surface represents the transmission efficiency in the conventional resonance WPT system with RTC, which is sensitive to the coupling parameters.

Apart from the efficiency stability in the working state, the idle power loss (corresponding to reflection loss) should be as low as possible in an idle state (without receiver terminals) to benefit intermittent wireless charging and reduce overall power consumption in the WPT system [62]. We conducted experiments to study the performance of the anti-resonance energy transfer system during idle and working states. Focusing on safety and energy saving, it is quite worthwhile to maintain a low-energy output in the idle state of the system. In fact, idle power loss is always an insurmountable problem in the conventional WPT scheme. However, for the anti-*PT*-symmetric system constructed by anti-resonance mode, this limitation can be naturally overcome. A comparison of idle power loss in the idle state, without receiver terminals for two types of WPT schemes with $R/r = 1.76$, is evaluated in Fig. 4(b). The calculated and measured idle power loss of the resonance (anti-resonance) WPT system with RTC (ATC) is marked by the solid line and symbols, respectively. It can be clearly seen that the idle power loss of the anti-resonance WPT near the working frequency is significantly smaller than the resonance case, which is conducive to the energy conservation and better security in practical applications.

Under the working state, the transfer efficiency of the synthetic anti-resonance, compared with the conventional resonance, WPT system is shown in Fig. 4(c). The calculated and measured results of the RTC (ATC) WPT system are shown by the cyan solid line (pink dashed line) and cyan stars (pink circles), respectively. Different from the RTC case in which the optimal working frequency is tracked with different radius ratios (for example, the working frequencies of the system with $R/r = $ 1.76 and 2.31 are selected as 198.3 kHz and 225 kHz, respectively), the working frequency for the synthetic anti-resonance system is always fixed at ${f}_0 = 225$ kHz. Moreover, the transfer efficiency of the novel third-order anti-*PT*-symmetric WPT system is relatively stable, while in the conventional resonance WPT system it declines sharply when the system corresponds to the weak coupling region, which is indicated by the shading region ($R/r > 1.98$). From these perspectives, such a third-order anti-*PT*-symmetric system, characterized by real eigenfrequency independent of radius ratio, can be seen as providing a highly efficient WPT strategy without frequency tracking. The optimized transmission efficiency of WPT is limited by the radiative loss of the system. With the reduction of the radius of the receiving coil, a larger radius ratio $R/r$ for energy transmission will be realized, but the transmission efficiency will decrease due to the increase in radiation loss. In fact, even if the matching condition is deviated (${\mathrm{\Delta }} \ne \sqrt 2 \kappa $), the level-pinning effect in the anti-*PT*-symmetric WPT system still supports a mode with slight dissipation at the resonance frequency $\omega = {{\mathrm{\omega }}}_0$, thus its efficient transfer efficiency is more robust than the previous WPT schemes with *PT*-symmetry, as shown in Fig. 4(d). For the fixed ${\mathrm{\Delta \ }} = {\mathrm{\ }}68.7{\mathrm{\ kHz}}$, the calculated and measured transfer efficiencies of the anti-*PT*-symmetric WPT systems as a function of $R/r$ are shown by the pink solid line and circles, respectively. For comparison, considering the optimized working frequency 198.3 kHz for the case of $r\ = \ $8.5 cm in the *PT*-symmetric system, the calculated and measured transfer efficiencies at the fixed frequency as a function of $R/r$ are shown by a solid line and stars, respectively. From Fig. 4(c) and (d), it can be clearly seen that whether the anti-*PT*-symmetric system deviates from the matching condition or not, it has higher transfer efficiency than traditional *PT*-symmetric WPT systems. Especially in the weak coupling region, taking $R/r$ = 4.29 as an example, the transfer efficiency of anti-*PT*-symmetric and *PT*-symmetric WPT systems that meet (deviate from) the matching condition is ∼0.73 (0.32) and 0.17 (0.10) respectively. Compared with *PT*-symmetric systems, the corresponding transfer efficiency of anti-*PT*-symmetric systems is improved nearly 4.3- (3.2) fold. Efficient WPT for multiple loads in the synthetic anti-resonance system can be seen in the Supplementary Data.

## CONCLUSIONS

In summary, the efficient third-order non-Hermitian WPT system with anti-*PT*-symmetry is theoretically proposed and experimentally fabricated in this work. Based on the level pinning of the anti-resonance structure, a synthetic anti-*PT*-symmetric WPT system with miniaturization and integration is experimentally fabricated for realizing stable and efficient WPT. Compared with conventional resonant WPT systems, it has been confirmed that anti-*PT*-symmetric WPT systems can achieve robust WPT for systems with different sizes of loads. The results agree with the theoretical prediction that the destructive interference between the detuning modes of the anti-resonance structure enables the position of the working frequency to be maintained. The results for third-order non-Hermitian WPT systems with anti-*PT*-symmetry not only provide a good platform to study non-Hermitian WPT with higher performance (higher transmission efficiency and lower standby power loss) than conventional resonance WPT schemes, but also pave the way for other applications in integrated photonics, like resonance imaging, high-sensitivity sensors and wireless communications.

## Supplementary Material

nwad172_Supplemental_File
