# Supplementary material for: Level pinning of anti-PT-symmetric circuits for efficient wireless power transfer
Source: Natl Sci Rev. 2023 Jun 14;11(1):nwad172. doi: 10.1093/nsr/nwad172 (PMC10727848; doi:10.1093/nsr/nwad172)
Supplement: nwad172_Supplemental_File [file nwad172_supplemental_file.docx]

**Supplementary** **Materials for “Level pinning of anti-*PT* symmetric circuits for efficient wireless power transfer”**

Zhiwei Guo^1^*, Fengqing Yang^1^, Haiyan Zhang^1^, Xian Wu^1^, Qiong Wu^1^, Kejia Zhu^2^, Jun Jiang^3^, Haitao Jiang^1^, Yaping Yang^1^, Yunhui Li^1^*, and Hong Chen^1^*

^1^ MOE Key Laboratory of Advanced Micro-structured Materials, School of Physics Sciences and Engineering, Tongji University, Shanghai 200092, China.

^2^ Department of Electrical Engineering, Tongji University, Shanghai 201804, China.

^3^ School of Automotive Studies, Tongji University, Shanghai 210804, China.

*Corresponding authors’ Emails:

[2014guozhiwei@tongji.edu.cn](mailto:2014guozhiwei@tongji.edu.cn); [liyunhui@tongji.edu.cn](mailto:liyunhui@tongji.edu.cn); [hongchen@tongji.edu.cn](mailto:hongchen@tongji.edu.cn).

[Section A: The schematic of conventional resonance WPT 1](#_Toc136706785)

[Section B: Level pinning of the third-order anti-*PT*-symmetric system 4](#_Toc136706786)

[(1) Level attraction due to the dissipative coupling in anti-resonance mode 4](#_Toc136706787)

[(2) Level repulsion due to the coherent coupling between the resonance modes in the anti-resonance and resonance structures 5](#_Toc136706788)

[(3) Hamiltonian of the third-order anti-*PT*-symmetric system 7](#_Toc136706789)

[(4) Level pinning realized by the competition between dissipative coupling and coherent coupling 8](#_Toc136706790)

[(5) The comparison of transfer efficiency for WPT systems with different transmitters 11](#_Toc136706791)

[(6) The comparison of transfer efficiency for WPT systems with multi-loads 13](#_Toc136706792)

[Section C: The details of anti-*PT*-symmetric system with synthetic ART coil 14](#_Toc136706793)

[(1) The third-order anti-*PT*-symmetry established based on the circuit theory 14](#_Toc136706794)

[(2) Eigenstate analysis for electromagnetic compatibility 18](#_Toc136706795)

[(3) Robustness of level pinning to loads with different power 21](#_Toc136706796)

[(4) Level pinning to efficient energy transfer with multi-loads 22](#_Toc136706797)

[(5) Experimental setup and details of measurement 26](#_Toc136706798)

[(6) Efficient transfer of WPT with ATC for actual power signal source (PSS) 27](#_Toc136706799)

[(7) Extended to higher order circuit model 31](#_Toc136706800)

# Section A: The schematic of conventional resonance WPT

For a standard resonance WPT scheme based on a general two-resonance system, as shown in Fig. S1(a). The resonant frequency of the transmitter coil and receiver coil is $\omega_{0}$. The signal input and output from the system from the source coil and load coil, respectively. The corresponding physical model is shown in Fig. S1(b). The input, reflected and output signals are represented by $S_{1+}$, $S_{1-}$, and $S_{2+}$, respectively. Especially, `the three-level scheme of the second-order non-Hermitian system is shown in Fig. S1(c). Similar to Eq. (1), the dynamic equation of this system in Fig. 1 can be written based on the coupled-mode theory [S1]

, (A1)

where $\gamma_{j}$ and $\Gamma_{j}$ ($j=1$,$2$) denote the radiative loss and dissipative loss of the harmonic modes $a_{j}=A_{j}e^{-i\omega t}$ in transmitter coil and receiver coil, respectively. $\kappa'=\sqrt{2}\kappa$ is the near-field coupling coefficient between two resonant coils. The power transfer efficiency of the two-resonance WPT system can be expressed as $\eta=\left|S_{21}\right|^{2}=\left|S_{2+}/S_{1+}\right|^{2}$, where output wave corresponds to $s_{2+}=\sqrt{2\gamma_{2}}a_{2}$. Especially, considering the zero reflected waves $S_{1-}=-S_{1,+}+\sqrt{2\gamma_{1}}a_{1}=0$, the same $\gamma_{1}=\gamma_{2}=\gamma^{'}=2\gamma$ and negligible loss ($\Gamma_{1}=\Gamma_{2}=0$) of two resonant coils, the transfer efficiency of the system can be simplified as

. (A2)

From Eq. (A2), the optimized transfer efficiency $\eta=1$ can be easily obtained for in the strong coupling region ($\kappa^{'}\geq\gamma^{'}$). Once the coupling strength is less than the critical value $\kappa_{c}^{'}=\gamma^{'}$, the transfer efficiency will be significantly reduced.

In addition, the effective Hamiltonian of the two-resonance WPT can be expressed as [S2]

$H=\left(\begin{matrix} \omega_{0}+i\gamma^{'} & \kappa^{'} \\ \kappa^{'} & \omega_{0}-i\gamma^{'} \end{matrix}\right)$. (A3)

From Eq. (A3), we can find that the non-Hermitian WPT system with the second-order *PT-*symmetry. The eigenfrequencies of the system can be solved as

. (A4)

Compared Eqs. (A4) with (A2), we can conclude that the stable power transfer state of the non-Hermitian WPT system with RTC is equivalent to a real eigenvalue of the effective Hamiltonian, which is consistent with the conclusion given in Eqs. (4) and (9) for the non-Hermitian WPT system with ATC.


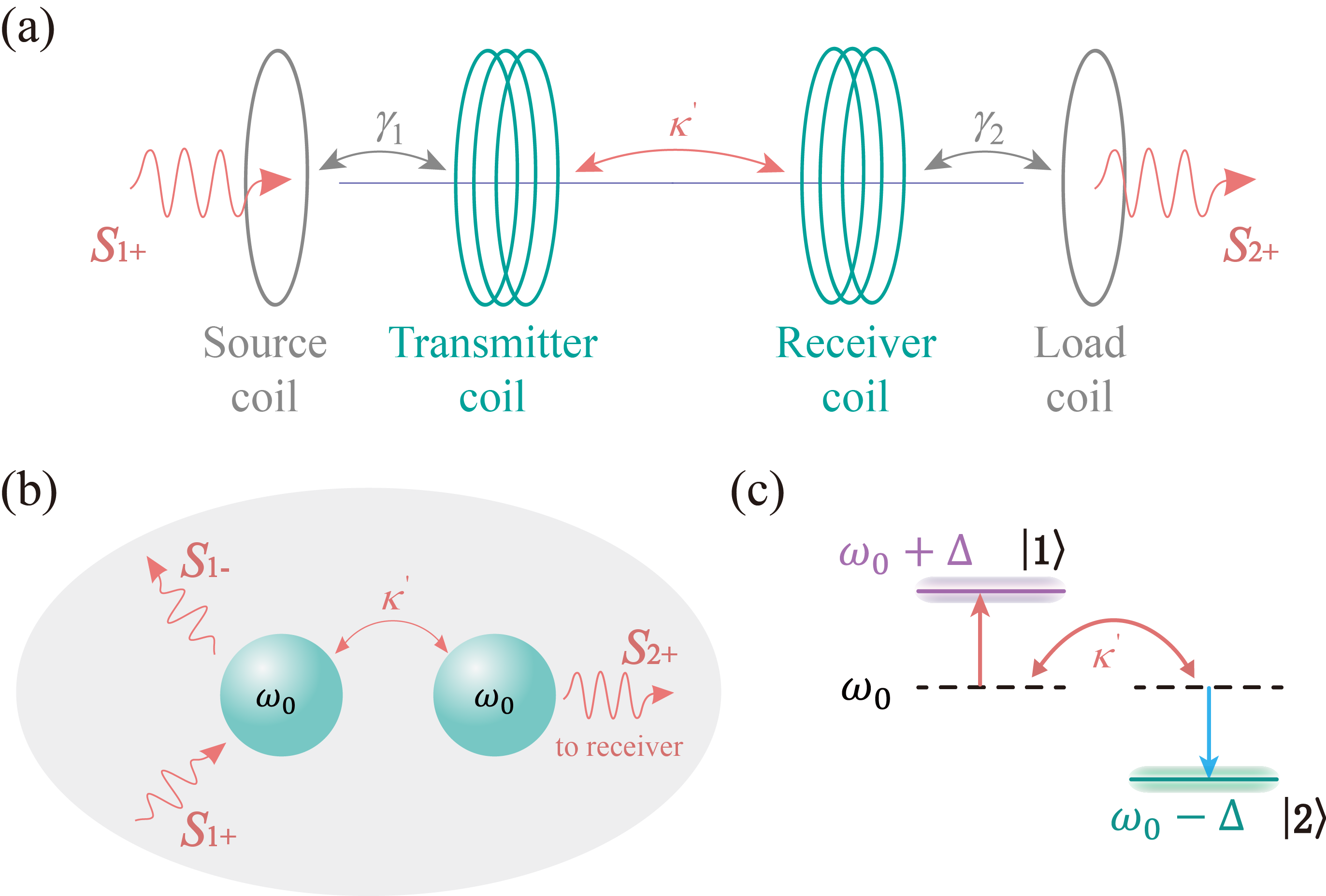


FIG. S1. **The conventional resonant WPT system**. (a) Scheme of a standard magnetic resonance WPT system. (b) Physical model of the non-Hermitian system consisting of a pair of coupled resonant modes with same resonant mode $\omega_{0}$. The near-field coupling strength between the resonant modes is represented by $\kappa$. (c) The corresponding three-level scheme. The frequency splitting ($\omega_{0}\pm\Delta$) is marked by the levels $\left. |1 \right\rangle$ and $\left. |2 \right\rangle$, respectively.

# Section B: Level pinning of the third-order anti-*PT*-symmetric system

## (1) Level attraction due to the dissipative coupling in anti-resonance mode

Considering the single anti-resonance mode in Fig. 1, the corresponding dynamic equation can be written based on the coupled-mode theory [S1]

, (B1)

where $\omega_{+}=\omega_{0}+\Delta$ and $\omega_{-}=\omega_{0}-\Delta$ are the two resonance frequencies of the anti-resonance mode. Here, we assume $\omega_{0}=5$,$\Delta=\sqrt{2}$ and $\gamma_{+}=\gamma_{-}=\gamma$, and ignore the intrinsic loss $\Gamma_{+}=\Gamma_{-}=0$. Similar to Eq. (1), we can obtain the effective Hamiltonian of the anti-resonance system. Figures S2(a) and S2(b) present the evolution of the real parts and imaginary parts of the eigenfrequencies as a function of dissipative coupling $\gamma$, respectively. It can be clearly seen that the bifurcated eigenvalues coalesce at EPs with $\Delta=\gamma$, which are marked by the gray arrows. Considering the case of energy transfer, we assume that the signal is input from mode ‘+’ and output from mode ‘-’. Then, we can also obtain the transmission spectra of the anti-resonance mode for the different normalized dissipative coupling $\gamma/\Delta$, as shown in Fig. S2(c). With the increase of dissipative coupling, the splitting modes gradually approach, and finally the mode coalescing occurs at EP. In order to show the level attraction effect in the anti-resonance structure more clearly, the analytical outgoing transmittance spectra for different dissipative coupling are shown in Fig. S2(d). From Fig. S2, we can clearly see that the modes the dissipative coupling can induce the level attraction effect in anti-resonance structure.


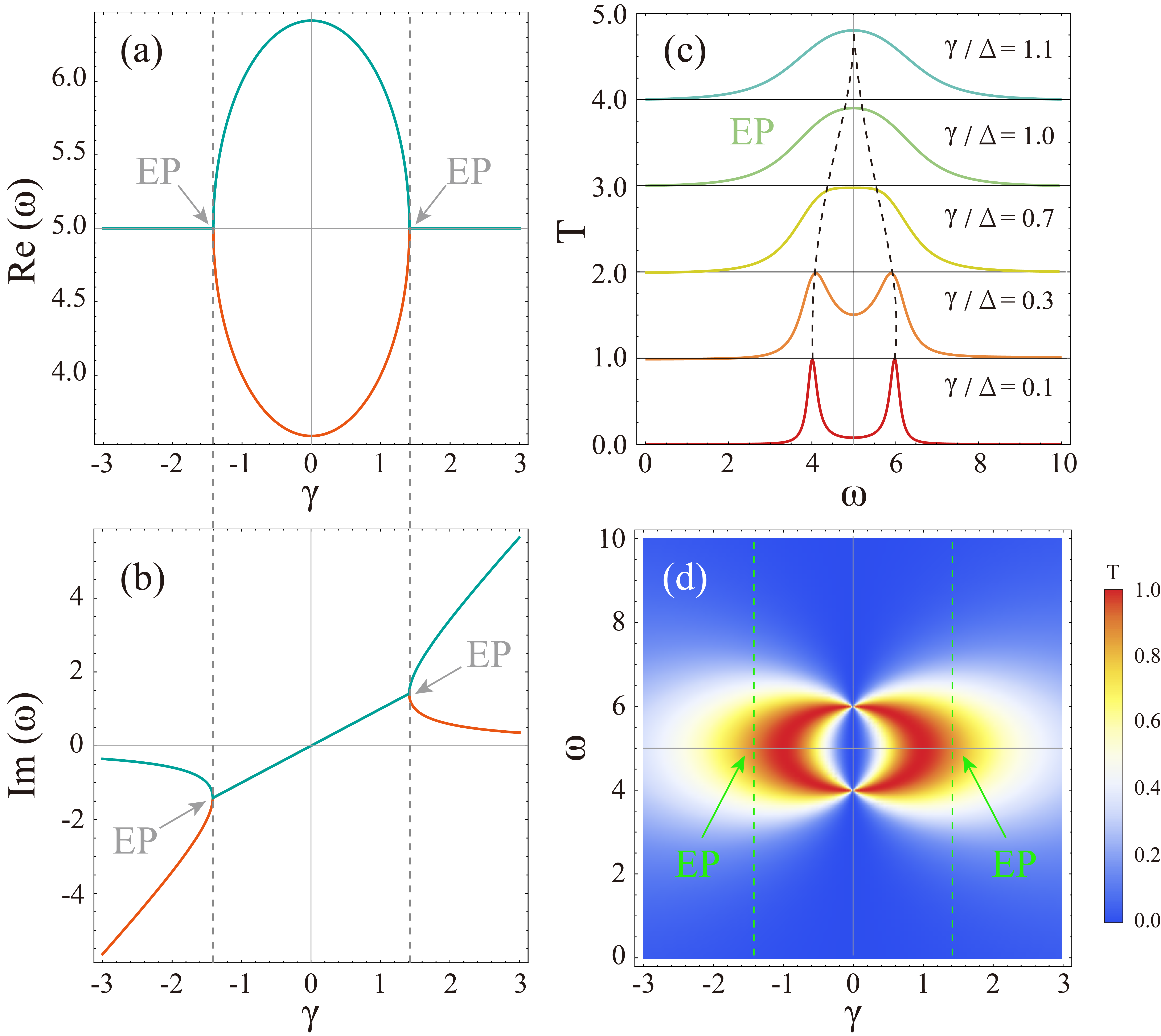


FIG. S2. **Level attraction in the anti-resonance mode with dissipative coupling**. Evolution of the real parts (a) and imaginary parts (b) of the eigenfrequencies in the anti-resonance system. The EPs are marked by the gray arrows. (c) Calculated transmission spectra of the anti-resonance mode for the different normalized dissipative coupling $\gamma/\Delta$. The transmission peaks are connected by black dashed lines to show the level attraction effect. (d) Analytical outgoing transmittance spectra for different dissipative coupling.

## (2) Level repulsion due to the coherent coupling between the resonance modes in the anti-resonance and resonance structures

In contrast to the level attraction of anti-resonance structure introduced in Sec. B(1), the level repulsion effect due to the coherent coupling between the resonance modes in the anti-resonance and resonance structures is shown in Fig. S3. Considering the anti-resonance and resonance structures in Fig. 1, the corresponding dynamic equation of the resonance modes $\omega_{j}$ ($j=+, -$) and $\omega_{0}$ can be written simply based on the coupled-mode theory [S1]

, (B2)

where $\kappa_{+0}=\kappa_{+}$ and $\kappa_{-0}=-\kappa_{-}$. Here, we take the mode $\omega_{+}=\omega_{0}+\Delta$ in anti-resonance structure for example, the corresponding real parts and imaginary parts of the eigenfrequencies as a function of coherent coupling $\kappa$ is shown in Figs. S3(a) and S3(b), respectively. The transmission spectrum of the system composed of one resonance mode of anti-resonance structure and the resonance mode in resonance structure for the different normalized coherent coupling $\kappa$ is shown in Fig. S3(c). With the increase of coherent coupling, the splitting modes gradually move away. In order to show the level repulsion effect realized by the resonance modes of the anti-resonance and resonance structures more clearly, the analytical outgoing transmittance spectra for different coherent coupling is shown in Fig. S3(d). Similar to Figs. 3(c) and 3(d), the transmittance spectra of the system composed of resonance mode ($\omega_{-}=\omega_{0}-\Delta$) in anti-resonance structure and resonance mode ($\omega_{0}$) in resonance structure are shown in Figs. 3(e) and 3(f). Therefore, from Fig. S3, the level repulsion effect due to the coherent coupling between the resonance modes in the anti-resonance and resonance structures is clearly demonstrated.


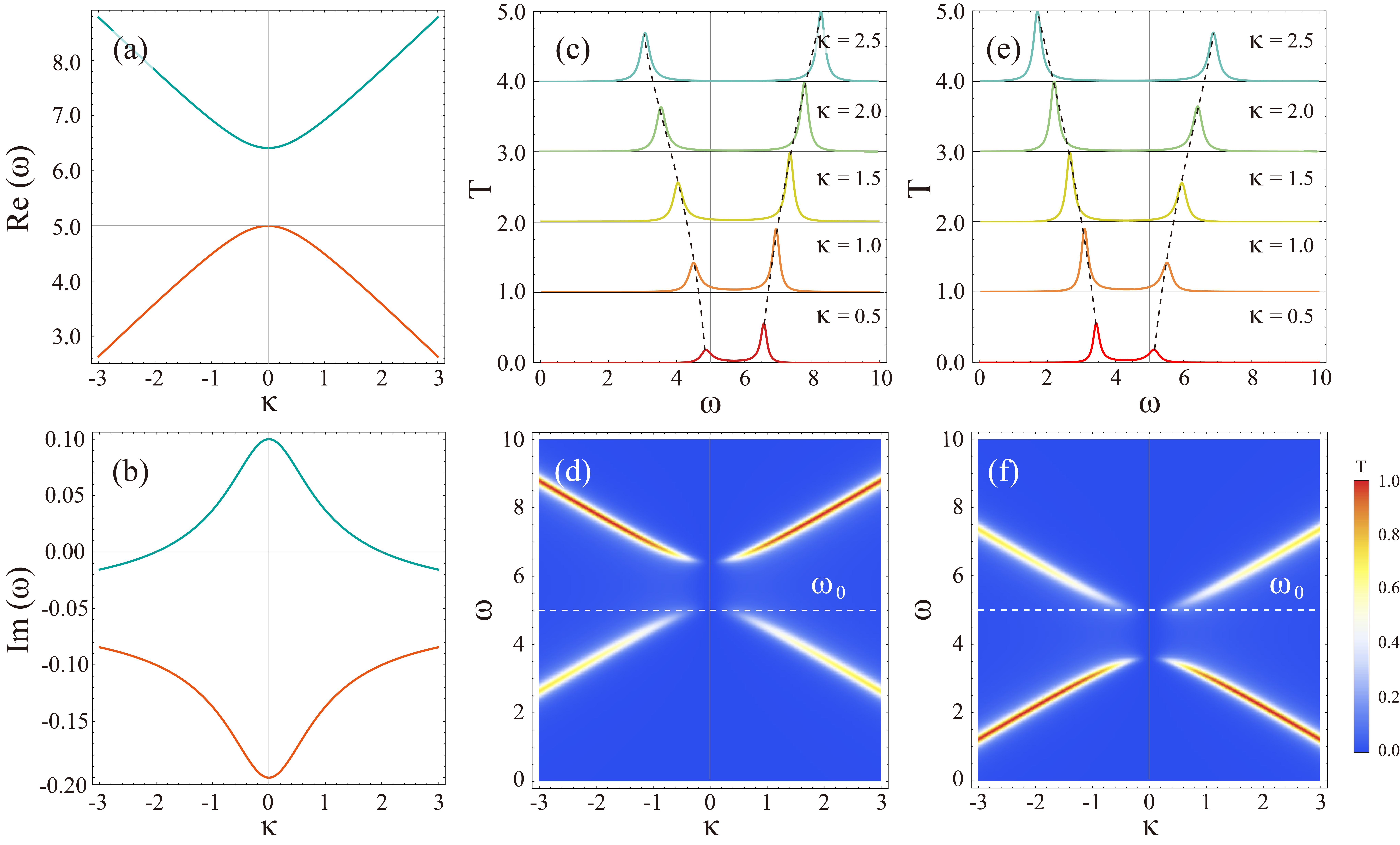


FIG. S3. **Level repulsion between the resonance modes of the anti-resonance and resonance structures**. Evolution of the real parts (a) and imaginary parts (b) of the eigenfrequencies in the system with resonance mode $\omega_{0}+\Delta$ and resonance mode $\omega_{0}$ in the anti-resonance structure and resonance structure, respectively. (c) Calculated transmission spectrum for the different coherent coupling $\kappa$ between resonance mode $\omega_{0}+\Delta$ and resonance mode $\omega_{0}$. The transmission peaks are connected by black dashed lines to show the level repulsion effect. (d) Analytical outgoing transmittance spectra for different coherent coupling between resonance mode $\omega_{0}+\Delta$ and resonance mode $\omega_{0}$. (e) (f) Similar to (c) (d), but for the level repulsion realized by the resonance mode $\omega_{0}-\Delta$ and resonance mode $\omega_{0}$.

## (3) Hamiltonian of the third-order anti-*PT*-symmetric system

Anti-*PT* symmetries have provided important guiding principles in the researches of non-Hermitian physics and revealed rich physics beyond conventional systems. For the third-order coupled system, the general form of the Hamiltonian can be written as

$\boldsymbol{H}=\left[ \begin{matrix} A+iB & C+iD & E+iF \\ C+iD & G+iH & I+iJ \\ E+iF & I+iJ & K+iL \end{matrix} \right]$, (B3)

where *A*, *G*, and *K* are the real frequencies. *B*, *H*, and *L* are the gain or damping rates, which can be positive or negative. $i$ is the imaginary unit. $C+iD$, $E+iF$ and $I+iJ$ represent the complex coupling strength, where *C*, *E*, and *I* are the coherent coupling strength, and $iD$, $iF$, and $iJ$ are the dissipative coupling strength. Without loss of generality, all the symbols are real numbers. If the coupled system is an anti-*PT*-symmetric non-Hermitian system, the Hamiltonian will satisfy {$\boldsymbol{H}_{\boldsymbol{APT}}$*, PT*}=0, that is

$(PT)\boldsymbol{H}_{\boldsymbol{APT}}(PT)^{-1}=-\boldsymbol{H}_{\boldsymbol{APT}}$*.* (B4)

Following the same formalism, one can obtain

$\left[ \begin{matrix} K-iL & I-iJ & E-iF \\ I-iJ & G-iH & C-iD \\ E-iF & C-iD & A-iB \end{matrix} \right]=\left[ \begin{matrix} -A-iB & -C-iD & -E-iF \\ -C-iD & -G-iH & -I-iJ \\ -E-iF & -I-iJ & -K-iL \end{matrix} \right]$, (B5)

and correspondingly, one will have $K-iL=-A-iB$, $I-iJ=-C-iD$, $E-iF=-E-iF$, and $G-iH=-G-iH$, which needs $K=-A$, $L=B$, $I=-C$, $J=D$, $E=$0, and $G=0$. As a result, the third-order anti-*PT*-symmetric system has a general form of the Hamiltonian as

$\boldsymbol{H}_{\boldsymbol{APT}}=\left[ \begin{matrix} A+iB & C+iD & iF \\ C+iD & iH & -C+iD \\ iF & -C+iD & -A+iB \end{matrix} \right]$. (B6)

Comparing Eqs. (B6) and (4), we can clearly see that the system studied in this work satisfies the anti-*PT*-symmetry with respect to the central frequency when $A=\Delta$, $B=\gamma$, $C=-\kappa$, $D=0$, $F=\gamma$, and $H=-2\gamma$.

## (4) Level pinning realized by the competition between dissipative coupling and coherent coupling

In Secs. B(1) and B(2), the level attraction and level repulsion have been demonstrated with the help of dissipative coupling and coherent coupling, respectively. Here, the novel level pinning realized by the competition between dissipative coupling and coherent coupling is introduced. According to the Hamiltonian given in Eq. (4), the eigenvalues of the anti-*PT*-symmetri*c* non-Hermitian system are

$\omega_{1}=-\frac{2^{1/3}A}{3(iB+\sqrt{4A^{3}-B^{2}})^{1/3}}+\frac{(iB+\sqrt{4A^{3}-B^{2}})}{3\times2^{1/3}}+\omega_{0}$, (B7)

$\omega_{2}=-\frac{(1+\sqrt{3}i)A}{3\times2^{2/3}(iB+\sqrt{4A^{3}-B^{2}})^{1/3}}+\frac{(1-\sqrt{3}i)(iB+\sqrt{4A^{3}-B^{2}})}{6\times2^{1/3}}+\omega_{0}$, (B8)

$\omega_{3}=-\frac{(1-\sqrt{3}i)A}{3\times2^{2/3}(iB+\sqrt{4A^{3}-B^{2}})^{1/3}}+\frac{(1+\sqrt{3}i)(iB+\sqrt{4A^{3}-B^{2}})}{6\times2^{1/3}}+\omega_{0}$, (B9)

where $A=3(4\gamma^{2}-\Delta^{2}-2\kappa^{2})$ and $B=54\gamma(\Delta^{2}-2\kappa^{2})$, respectively. The level pinning of the anti-*PT*-symmetric non-Hermitian system can be analytically demonstrated. Considering $x={(iB+\sqrt{4A^{3}-B^{2}})}^{1/3}$, we can easily obtain

$\left| x \right|^{2}=\left| iB+\sqrt{4A^{3}-B^{2}} \right|^{2/3}=\left( \sqrt{B^{2}+\left( \sqrt{4A^{3}-B^{2}} \right)^{2}} \right)^{2/3}=4^{1/3}A$. (B10)

As a result, the difference between the real part of the eigenvalue $\omega_{1}$ and the resonance frequency $\omega_{0}$ can be simplified as

$\mathrm{Re}\left( \omega_{1} \right)-\omega_{0}=-\frac{2^{1/3}A}{3}\cdot\frac{Re\left( x \right)}{\left| x \right|^{2}}+\frac{Re\left( x \right)}{3\cdot2^{1/3}}=-\frac{2^{1/3}A}{3}\cdot\frac{Re\left( x \right)}{4^{1/3}A}+\frac{Re\left( x \right)}{3\cdot2^{1/3}}=0$. (B11)

Therefore, the level pinning locked at the resonant frequency due to the competing couplings will always happen in the anti-*PT*-symmetric non-Hermitian system.

From Eq. (B7), we can determine that a pure real eigenfrequency of $\omega_{1}=\omega_{0}$ is enabled when the condition $\Delta=\sqrt{2}\kappa$ is satisfied. In order to more intuitively display the level pinning effect in this ideal anti-*PT*-symmetric non-Hermitian WPT system, we show the evolution of the real and imaginary parts of the eigenfrequencies as a function of dissipative coupling and coherent coupling in Figs. S4(a) and S4(b), respectively. It can be clearly seen that the locking of eigenfrequency occurs at the resonance frequency $\omega=\omega_{0}$, which is independent of other parameters of the system. On the Riemannian surface composed of three parameters ($\omega$,$\gamma$, and $\kappa$), we can clearly see a plane $\omega=\omega_{0}$ independent of the coupling coefficients $\gamma$ and $\kappa$. Especially, the exceptional lines (ELs) connected by EPs with $\kappa=\gamma$ are marked by the red dashed lines. The stable green plane ($\omega=\omega_{0}$) comes from the competition between dissipative coupling and coherent coupling in the system composed of anti-resonance and resonance modes. In addition, we study the robustness of level pinning in the general anti-*PT*-symmetric non-Hermitian system that cannot strictly guarantee $\Delta=\sqrt{2}\kappa$ all the time. Without losing any generality, setting $\gamma=1$, the evolution of the real parts and imaginary parts of the eigenfrequencies as a function of detuning factor and coherent coupling are shown in Figs. S4(c) and S4(d), respectively. Interestingly, although the imaginary parts of the eigenvalues are not always equal to zero in this general anti-*PT*-symmetric non-Hermitian system, real part of the eigenvalue $\omega=\omega_{0}$ is always locked at the resonance frequency due to the competition of two kinds of coupling. Furthermore, figure S5 gives the phase diagrams for the anti-*PT*-symmetric non-Hermitian system with different detuning factor. We can clearly see that the real part of first eigenvalue $\omega_{1}$ is always locking at the resonant frequency due to the competing couplings. Especially, the imaginary parts of the eigenvalue $\omega_{1}$ is zero for the ideal case with $\Delta=\sqrt{2}\kappa$. However, when the ideal condition $\Delta=\sqrt{2}\kappa$ is not satisfied, the imaginary part of the eigenvalues will not be zero. Even for non-ideal cases that the matching condition is deviated $\Delta\neq\sqrt{2}\kappa$, it is not a pure real eigenvalue at resonance frequency $\omega=\omega_{0}$, and there will be a loss that will lead to a slight transfer efficiency reduction. Nevertheless, because there is an intrinsic mode in the anti-*PT*-symmetric system, compared with the traditional WPT system, it still has higher transmission efficiency when the operational condition changes.


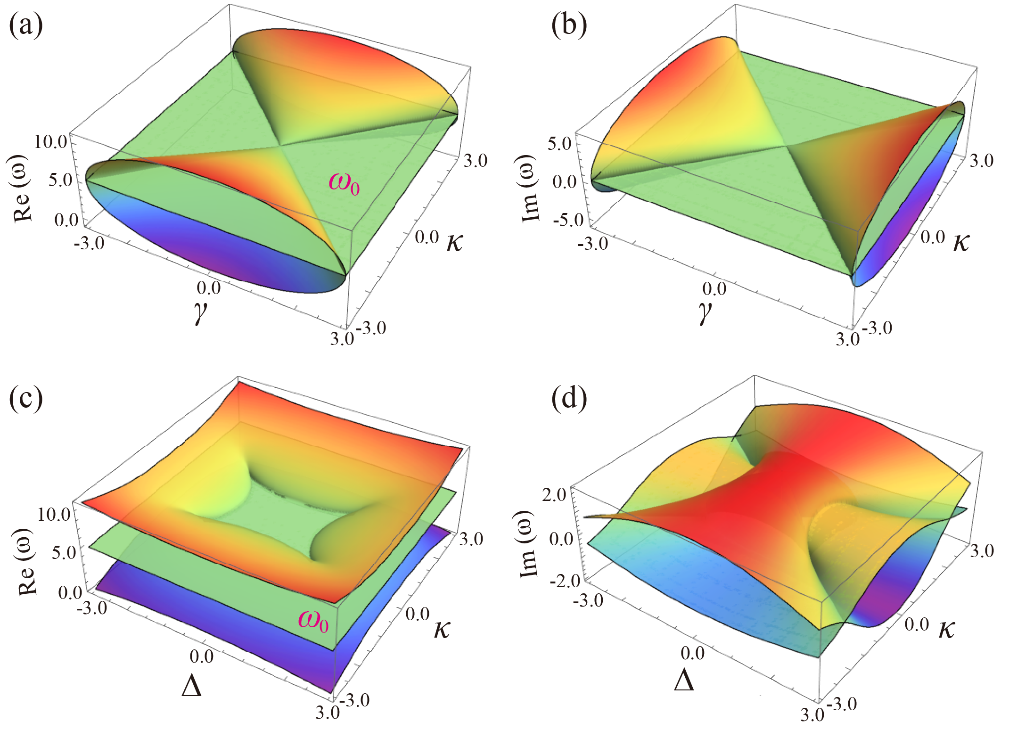


FIG. S4. **Robust level pinning due to the competing couplings**. (a) (b) The level pinning in an ideal anti-*PT*-symmetric non-Hermitian WPT system with $\Delta=\sqrt{2}\kappa$. Evolution of the real (a) and imaginary (b) parts of the eigenfrequencies as a function of dissipative coupling ($i\gamma$) and coherent coupling ($\kappa$) in the hybrid system composed of anti-resonance and resonance modes. (c) (d) Similar to (a) (b), but for the evolution of the eigenfrequencies as a function of detuning factor ($\Delta$) and coherent coupling ($\kappa$) in general anti-*PT*-symmetric non-Hermitian WPT system with $\gamma=1$. The green plane represents $\omega=\omega_{0}$.


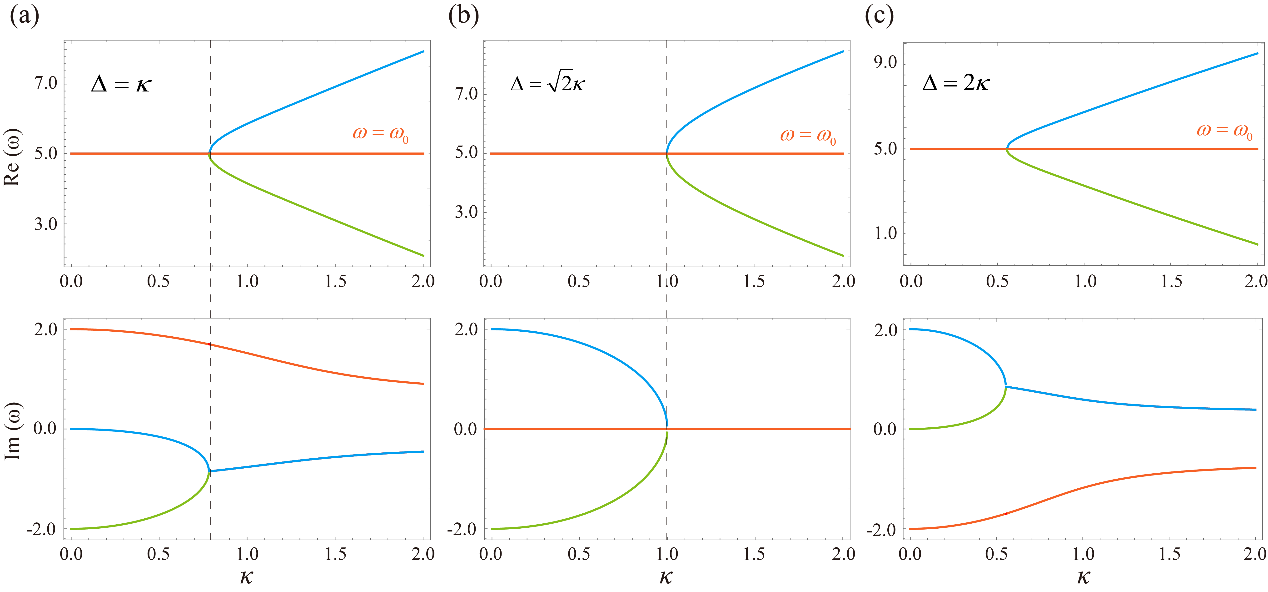


FIG. S5. **Level pinning in different anti-*PT*-symmetric non-Hermitian systems with** $\gamma=1$. (a) Eigenfrequencies for the anti-*PT*-symmetric non-Hermitian system with $\Delta=\kappa$. (b) (c) Same as (a), but for the anti-*PT*-symmetric non-Hermitian system with $\Delta=\sqrt{2}\kappa$ and $\Delta=2\kappa$, respectively.

## (5) The comparison of transfer efficiency for WPT systems with different transmitters

Although there are anti-*PT*-symmetry and anti-*PT*-symmetry breaking phases identified by EPs in anti-*PT*-symmetric systems, the eigenvalue of the level pinning position is always pure real in both strong and weak coupling regions, and can be used for efficient and stable energy transfer. Therefore, anti-*PT*-symmetric systems can overcome the decrease in transmission efficiency caused by weak coupling in *PT*-symmetric systems. The comparison of the *PT-*symmetric and anti-*PT-*symmetric systems with RTC and ATC respectively under the same parameters is shown in Fig. S6. Without losing any generality, setting $\gamma=1$ and $\omega_{0}=5$, the transfer efficiency of the lossless and lossy conventional *PT-*symmetric WPT system are shown in Figs. S6(a)-S6(c), respectively. Especially, the transfer efficiency of this well-known resonance WPT system with negligible intrinsic loss is shown in Fig. S6(a). Due to the near-field coupling effect, the working frequency of the resonant system will split in strong coupling region, and the optimized transfer efficiency can be realized at $\omega_{+}=\omega_{0}+\delta\omega$ and $\omega_{-}=\omega_{0}-\delta\omega$, where the frequency detuning is $\delta\omega=\sqrt{\kappa^{2}-\gamma^{2}}$. In addition, the transfer efficiency decreases rapidly when the coupling strength is smaller than the critical value $\kappa_{c}$, and this critical case corresponds to the EP_RTC_ of the non-Hermitian WPT system, which is marked by the cyan arrow in Fig. S6(a). Similar results can be found in the lossy condition. The transfer efficiency of the conventional *PT*-symmetric WPT system with $\Gamma=0.05$ and $\Gamma=$ 0.1 are shown in Figs. S6(b) and S6(c), respectively. Therefore, in addition to the unstable working frequency, the low transfer efficiency in weak coupling region is also an obvious disadvantage of the conventional resonance WPT scheme. However, in the anti-*PT*-symmetric WPT system, it should be emphasized that except for two split frequencies, the transmission efficiency of working frequency $\omega=\omega_{0}$ is always optimized with different coupling strengths in the losses system. In other words, when the working frequency is selected at the pinning mode $\omega_{0}$, the transmission of the system is very stable and there is no need for frequency tracking. Moreover, figure S6(d) shows that the transmission efficiency of $\omega_{0}$ can remain optimal even when it exceeds the critical value—EP_ATC_. In other words, the use of anti-*PT*-symmetric systems for energy transfer is not affected by the coupling strength (transfer distance or the size ratio of the receiver to the transmitter) when ignoring the intrinsic loss of the system. In fact, although the transmission efficiency decrease for the actual system with intrinsic loss, the anti-*PT*-symmetric system still has the characteristics of working frequency locking and higher transmission efficiency at the weak coupling region, as shown in Figs. S6(e) and S6(f).


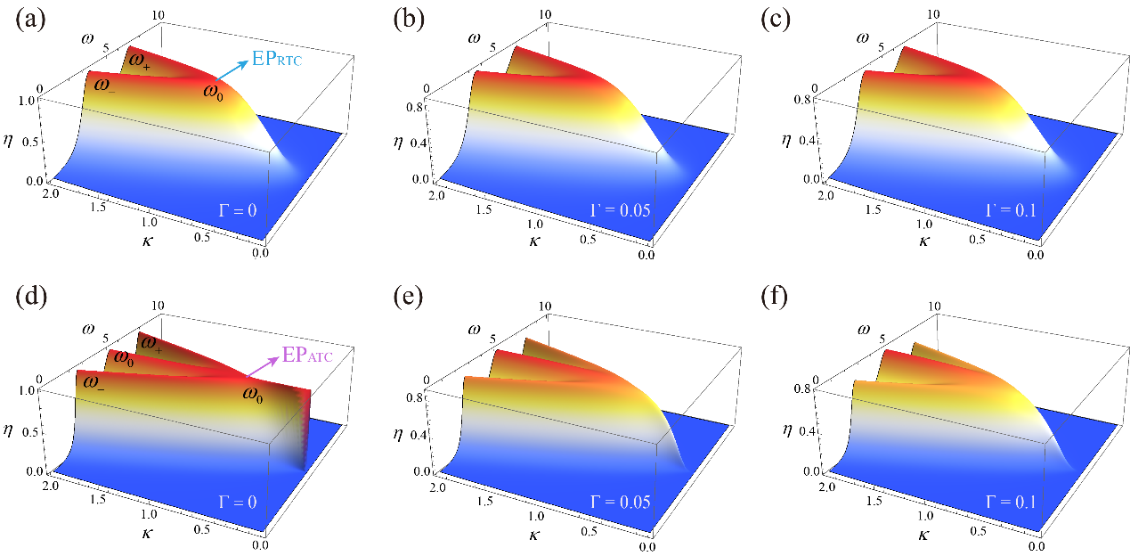


FIG. S6. **The comparison of transfer efficiency for two types of WPT systems**. The transfer efficiency of the conventional *PT*-symmetric WPT system with RTC: (a) lossless case $\Gamma=0$, (b) lossy case $\Gamma=0.05$, and (c) lossy case $\Gamma=0.1$, respectively. (d)-(f) Similar to (a)-(c), but for the transfer efficiency of the anti-*PT*-symmetric WPT system with ATC. The positions of EPs in *PT*-symmetric and anti-*PT*-symmetric non-Hermitian systems are marked by ${EP}_{RTC}$ and ${EP}_{ATC}$, respectively.

## (6) The comparison of transfer efficiency for WPT systems with multi-loads

The level pinning in anti-*PT-*symmetric system is robust against to the change of operational conditions, which can also be used to realize efficient multi-load energy transfer. From the single load case in Figs. S7(a) and S7(b), it can be found that the transfer efficiency of the conventional *PT*-symmetric WPT system strongly depends on the operational condition. However, the stable WPT can be realized in anti-*PT-*symmetric system with the aid of level pinning effect. As a result, considering two loads with different dissipative loss and/or coupling strength, the comparison of transfer efficiency at resonance frequency $\omega_{0}$ in two kinds of WPT systems is shown in Figs. S7(c) and S7(d). It can be seen that the transfer efficiency of different loads in an anti-*PT*-symmetric system is always higher than that of a *PT*-symmetric system.


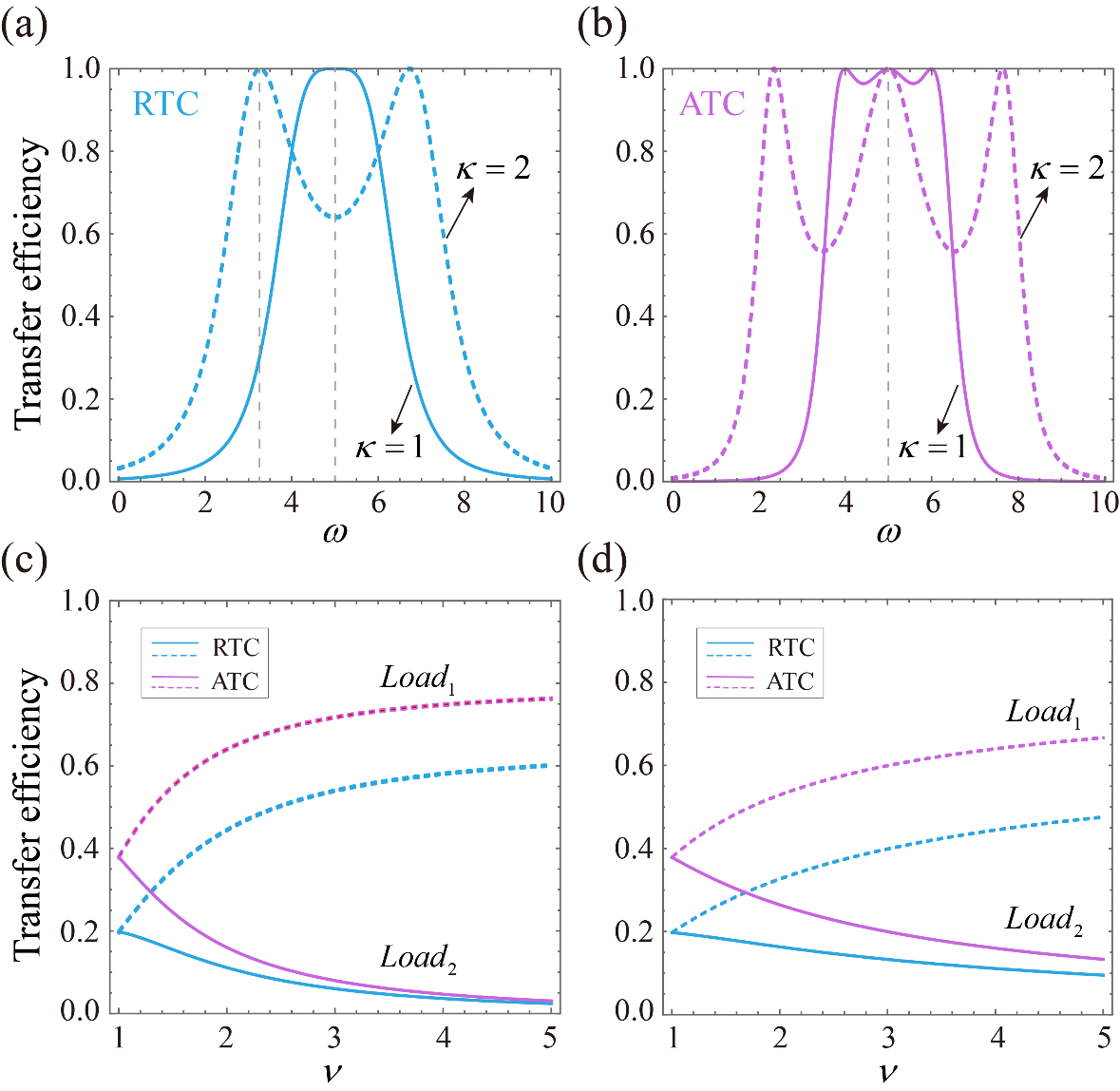


FIG. S7. **The comparison of transfer efficiency for multi-loads WPT**. (a) The transfer efficiency of the conventional *PT*-symmetric WPT systems with a single load, where $\kappa=$1 and $\kappa=$2 cases are marked by the solid line and dashed line, respectively. (b) Same as (a), but for the anti-*PT*-symmetric WPT systems. (c) The comparison of transfer efficiency in two kinds of WPT systems with two loads, where $\gamma_{1}=1$, $\gamma_{2}=1$, $\kappa_{1}=$2, and $\kappa_{2}=\kappa_{1}/\nu$. (d) Same as (c), but for the two loads with $\gamma_{1}=1$, $\gamma_{2}=\gamma_{1}/\nu$, $\kappa_{1}=$2, and $\kappa_{2}=\kappa_{1}/\nu$.

# Section C: The details of anti-*PT*-symmetric system with synthetic ART coil

## (1) The third-order anti-*PT*-symmetry established based on the circuit theory

Considering the electrodynamic potential of an AC source $U=-I_{1}Z$, the Kirchhoff’s equations of ATC in Fig. S8(a) can be expressed as

$\begin{matrix} (i\omega L_{1}+1/i\omega C_{1}+1/i\omega C_{0}-Z)I_{1}+I_{2}/i\omega C_{0}=0 \\ I_{1}/i\omega C_{0}+(i\omega L_{2}+1/i\omega C_{2}+1/i\omega C_{0})I_{2}=0 \end{matrix}$, (C1)

where $I_{1}$ and $I_{2}$ are the electric current along different direction, respectively. $-Z$ denotes the impedance of the source, which can be seen as the effective gain. To simplify the system, we consider the same inductance $L_{1}=L_{2}=L$ and the same capacitor $C_{2}=C_{1}$. In addition, we assume$1/C=1/C_{1}+1/C_{0}$, $\omega_{0}=1/\sqrt{LC}$and make an appropriate approximation $\omega^{2}-\omega_{0}^{2}\approx2\omega(\omega-\omega_{0})$. Considering the amplitude $a_{n}=-iL_{n}dI_{n}/d\tau$ ($n=1,2$) and $\tau=\omega t$, the dynamic equation of ATC can be obtained

$\begin{matrix} (\omega_{0}-\omega-Zi/2L)a_{1}+(1/2\omega C_{0}L)a_{2}=0 \\ (1/2\omega C_{0}L)a_{1}+(\omega_{0}-\omega)a_{2}=0 \end{matrix}$. (C2)

It should be emphasized that the loss of distributed inductances can be ignored because it is quite small compared with the lumped resistances. In order to connect the Kirchhoff’s equations of the circuit with the coupled mode theory, we can assume the effective gain and the effective coupling coefficient of the ATC are $\gamma_{0}=-Z/2L$ and $\kappa=1/2\omega C_{0}L$, respectively. Especially, considering a load whose impedance is equal to$Z$, the ATC can be considered as an ideal non-Hermitian system satisfying two-order *PT-*symmetry [S1].

After introducing the synthetic ATC, we will further introduce the novel synthetic anti-resonance WPT system. The corresponding effective circuit model of the synthetic third-order anti-*PT*-symmetric WPT is shown in Fig. S8(b). Similar to the consideration for the ATC, we also assume that $L_{3}=L$, $C_{3}=C$, and $R=Z$. In this case, the Kirchhoff’s equations can be expressed as

$\begin{matrix} (i\omega L+1/i\omega C_{1}+1/i\omega C_{0}-Z)I_{1}+I_{2}/i\omega C_{0}=0 \\ I_{1}/i\omega C_{0}+(i\omega L+1/i\omega C_{1}+1/i\omega C_{0})I_{2}+i\omega MI_{3}=0 \\ i\omega MI_{2}+(i\omega L+1/i\omega C+Z)I_{3}=0 \end{matrix}$, (C3)

where $M=\xi L$ denotes the mutual inductance between the synthetic ATC and RRC. $\xi=C/C_{0}$ is the coupling factor for different loads. Similar to Eq. (C1), the equations Eq. (C3) can be rewritten as

$\begin{matrix} (\omega_{0}-\omega-Zi/2L)a_{1}+(1/2\omega C_{0}L)a_{2}=0 \\ (1/2\omega C_{0}L)a_{1}+(\omega_{0}-\omega)a_{2}-(M\omega/2L)a_{3}=0 \\ -(M\omega/2L)a_{2}+(\omega_{0}-\omega+Zi/2L)a_{3}=0 \end{matrix}$. (C4)

From Eq. (C4), we can obtain the dynamic equation of the anti-resonance WPT system

$\begin{matrix} \frac{da_{1}}{dt}=(-i\omega_{0}-Z/2L)a_{1}-i(1/2\omega C_{0}L)a_{2} \\ \frac{da_{2}}{dt}=-i\omega_{0}a_{2}-i(1/2\omega C_{0}L)a_{1}+i(M\omega/2L)a_{3} \\ \frac{da_{3}}{dt}=(-i\omega_{0}+Z/2L)a_{3}+i(M\omega/2L)a_{2} \end{matrix}$. (C5)

Under a suitable unitary transformation

$a_{1}=(a_{+}+a_{-})/\sqrt{2}, a_{2}=(a_{+}-a_{-})/\sqrt{2}$, and $a_{3}=a_{0}$, (C6)

the dynamic equation of the anti-*PT*-symmetric system near the working frequency ($\omega=\omega_{0}$) becomes

$\begin{matrix} \frac{da_{+}}{dt}=-i(\omega_{0}+\frac{1}{2\omega C_{0}L}-\frac{Z}{4L}i)a_{+}-\frac{Z}{4L}a_{-}+\frac{iM\omega_{0}}{2\sqrt{2}L}a_{0} \\ \frac{da_{0}}{dt}=-i(\omega_{0}+\frac{Z}{2L}i)a_{0}+\frac{iM\omega_{0}}{2\sqrt{2}L}(a_{+}-a_{-}) \\ \frac{da_{-}}{dt}=-i(\omega_{0}-\frac{1}{2\omega C_{0}L}-\frac{Z}{4L}i)a_{-}-\frac{Z}{4L}a_{+}-\frac{iM\omega_{0}}{2\sqrt{2}L}a_{0} \end{matrix}$. (C7)

Then we define$\Delta=1/2\omega C_{0}L$ and $\gamma_{0}=-Z/2L$, the dynamic equation derived from the Kirchhoff’s equations are

$\left(\begin{matrix} \omega_{0}+\Delta+\frac{\gamma_{0}}{2}i & -\frac{\Delta}{\sqrt{2}} & \frac{\gamma_{0}}{2}i \\ -\frac{\Delta}{\sqrt{2}} & \omega_{0}-i\gamma_{0} & \frac{\Delta}{\sqrt{2}} \\ \frac{\gamma_{0}}{2}i & \frac{\Delta}{\sqrt{2}} & \omega_{0}-\Delta+\frac{\gamma_{0}}{2}i \end{matrix}\right)\left(\begin{matrix} a_{+} \\ a_{0} \\ a_{-} \end{matrix}\right)=\omega\left(\begin{matrix} a_{+} \\ a_{0} \\ a_{-} \end{matrix}\right)$ (C8)

The equivalent Hamiltonian of the system can be written as

$H=\left(\begin{matrix} \omega_{0}+\Delta+i\gamma& -\kappa& i\gamma\\ -\kappa& \omega_{0}-2i\gamma& \kappa\\ i\gamma& \kappa& \omega_{0}-\Delta+i\gamma\end{matrix}\right)$, (C9)

where $\gamma=\gamma_{0}/2$ and$\kappa=\Delta/\sqrt{2}$. From Eq. (C9), we can clearly see that the anti-*PT*-symmetry of the third-order non-Hermitian system about the center $\omega_{0}$ is verified $(PT)H(PT)^{-1}=PH^{*}P=-H$.


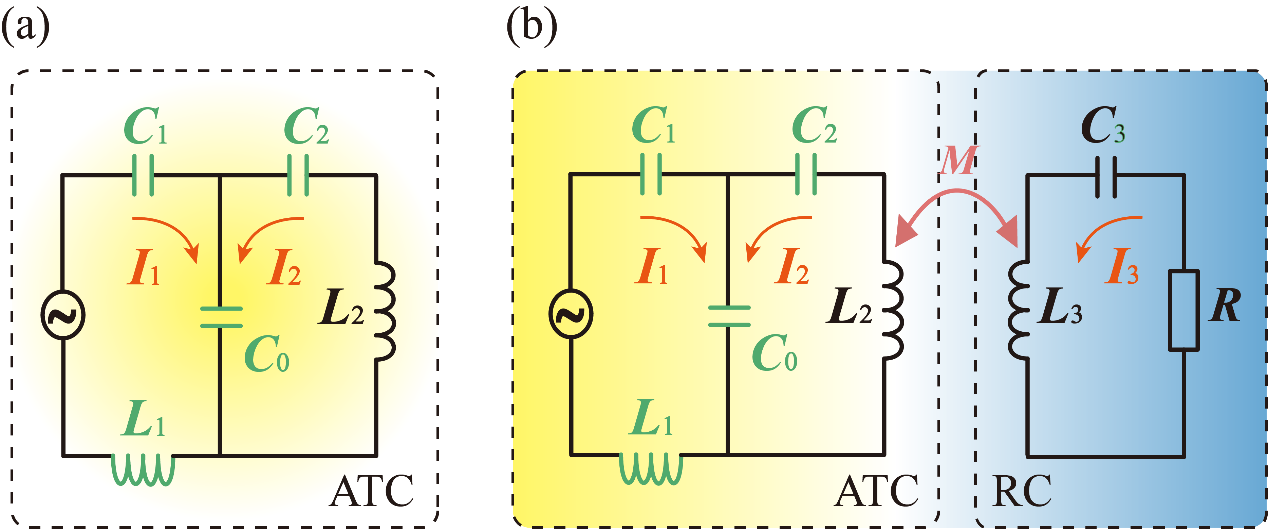


FIG. S8. **The effective circuit model of the synthetic third-order non-Hermitian WPT system with anti-*PT*-symmetry**. (a) The single ATC. (b) The coupled system between ATC and RRC.

From Eq. (C9), we can also obtain the evolution of real and imaginary parts of eigenfrequencies in the synthetic third-order anti-*PT*-symmetric non-Hermitian system when the radius ratio between ATC and RRC changes, as shown in Figs. S9(a) and S9(b), respectively. For comparison, the phase diagram of conventional resonance WPT with RTC under same parameters is also given by the dashed lines. As introduced in the main text, the EP associated radius ratio in the ATC system is larger than that of the RTC system under the same parameters. Moreover, the comparison of the real part of eigenfrequencies between experimental measurement and theoretical calculation is shown in Fig. 3(d) of the main text.


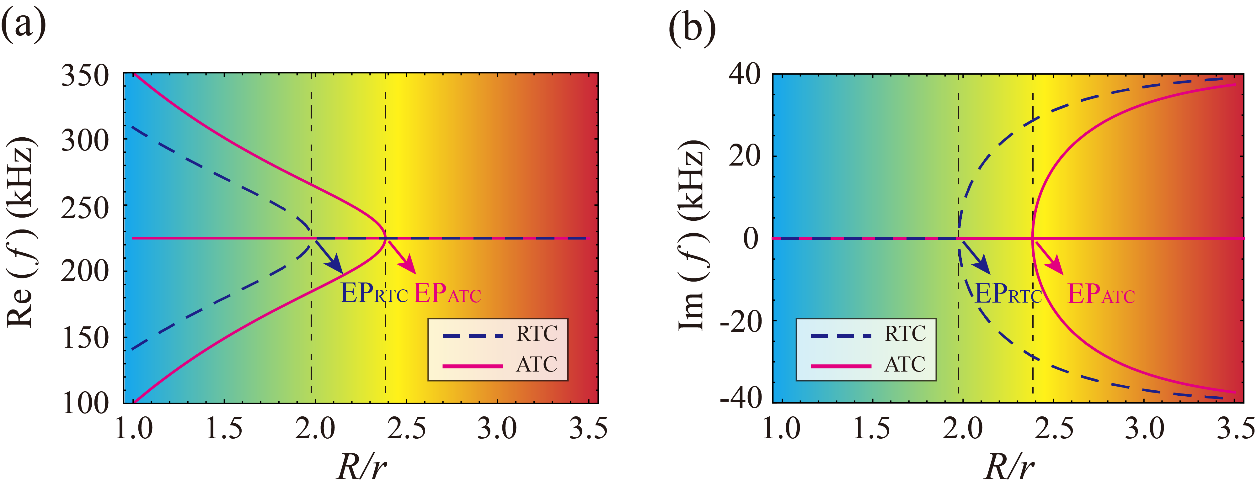


FIG. S9. **Phase diagrams for the synthetic third-order anti-*PT*-symmetric non-Hermitian system**. (a) Evolution of the real parts of eigenfrequencies in the synthetic third-order anti-*PT*-symmetric non-Hermitian system as a function of the radius ratio of synthetic ATC and RRC, which is marked by the pink solid line. For comparison, the phase diagram of conventional RS is also given by the blue dashed line. (b) Similar to (a), but for the evolution of the imaginary parts of eigen-frequencies. The EPs of the conventional system with RTC and the novel system with ATS are marked by the ${EP}_{RTC}$ and ${EP}_{ATC}$, respectively.

|  | $r (cm)$ | | | | | | | |
| --- | --- | --- | --- | --- | --- | --- | --- | --- |
|  | $5$ | $7$ | 9 | 11 | 13 | 15 | 17 | 19 |
| $\kappa_{0}$ (kHz) | 5.10 | 8.89 | 15.10 | 19.81 | 28.71 | 35.55 | 53.00 | 57.20 |
| $C_{0}$ (nF) | 112.51 | 64.54 | 38.00 | 28.96 | 19.99 | 16.14 | 10.83 | 10.03 |

**Table S1.** In the synthetic third-order anti-*PT*-symmetric WPT system, the relationship between coupling strength and the lumped bypass capacitance when the RRC with different radius, where $\kappa_{0}=\kappa/2\pi$.

## (2) Eigenstate analysis for electromagnetic compatibility

The magnetic field distributions of WPT system can be determined by the eigenstates of the transformed effective Hamiltonian in the position space. For the conventional resonance WPT in Fig. S1, and the eigenstates of the high frequency ($\omega_{2\div}=\omega_{0}+\sqrt{\kappa^{2}-\gamma_{0}^{2}}$) and low frequency ($\omega_{2-}=\omega_{0}-\sqrt{\kappa^{2}-\gamma_{0}^{2}}$) can be obtained directly from the Hamiltonian Eq. (A3) as

$X_{2-}=\left(\frac{\gamma i-\sqrt{\kappa^{2}-\gamma^{2}}}{\kappa},1\right),X_{2+}=\left(\frac{\gamma i+\sqrt{\kappa^{2}-\gamma^{2}}}{\kappa},1\right)$. (C10)

From Eq. (C10), it can be easily determined that the in the working state (*PT*-symmetric phase $\kappa>\gamma_{0}$), the density distributions of the coils correspond to *X*_2-_ = (1, 1) and *X*_2+_ = (1, 1), respectively. Therefore, the electromagnetic compatibility in the conventional resonance WPT system is inevitable because the amplitude distribution in the RTC is not negligible. However, this challenge can be well solved by the synthetic anti-*PT*-symmetric symmetric non-Hermitian WPT system. The transformed effective Hamiltonian in the position space of the synthetic anti-*PT*-symmetric non-Hermitian WPT system can be obtained under a suitable unitary transformation

$a_{+}=(a_{1}+a_{2})/\sqrt{2}, a_{-}=(a_{1}-a_{2})/\sqrt{2}$, and $a_{0}=a_{3}$. (C11)

As a result, the dynamics in the system can be represented as $H_{T}V=\omega V$, where $V=(a_{1},a_{2},a_{3})^{T}$, and the transformed effective Hamiltonian can be expressed as

$H_{T}=\left(\begin{matrix} \omega_{0}+i\gamma_{0} & \frac{1}{2\omega_{0}C_{0}L} & 0 \\ \frac{1}{2\omega_{0}C_{0}L} & \omega_{0} & -\frac{M\omega_{0}}{2L} \\ 0 & -\frac{M\omega_{0}}{2L} & \omega_{0}-i\gamma_{0} \end{matrix}\right)=\left(\begin{matrix} \omega_{0}+2i\gamma& \sqrt{2}\kappa& 0 \\ \sqrt{2}\kappa& \omega_{0} & -\sqrt{2}\kappa\\ 0 & -\sqrt{2}\kappa& \omega_{0}-2i\gamma\end{matrix}\right)$. (C12)

The corresponding eigenstates of transformed Hamiltonian $H_{T}$ at $\omega_{10}=\omega_{0}, \omega_{1-}=\omega_{0}-2\sqrt{\kappa^{2}-\gamma^{2}}$ and $\omega_{1+}=\omega_{0}+2\sqrt{\kappa^{2}-\gamma^{2}}$ are

$\begin{matrix} X_{10}=\left(1,-\frac{\sqrt{2}\gamma}{\kappa}i,1\right), \\ X_{1-}=\left(\frac{-\kappa^{2}+2\gamma^{2}+2\gamma i\sqrt{\kappa^{2}-\gamma^{2}}}{\kappa^{2}},\frac{-\sqrt{2}\gamma i-\sqrt{2\kappa^{2}-2\gamma^{2}}}{\kappa},1\right) \\ X_{1+}=\left(\frac{-\kappa^{2}+2\gamma^{2}-2\gamma i\sqrt{\kappa^{2}-\gamma^{2}}}{\kappa^{2}},\frac{-\sqrt{2}\gamma i+\sqrt{2\kappa^{2}-2\gamma^{2}}}{\kappa},1\right) \end{matrix}$, (C13)

respectively. Especially, since there is no leakage field in the lumped circuit elements, we only analyze the eigenstates of the transmitting and receiving coils with mutual inductance coupling. Then the eigenstates of transformed Hamiltonian $H_{T}$ in the position space at $\omega_{1-},\omega_{10}$ and $\omega_{1+}$, can be expressed as

$\begin{matrix} X_{10}=\left(-\frac{\sqrt{2}\gamma}{\kappa}i,1\right), \\ X_{1-}=\left(\frac{-\sqrt{2}\gamma i-\sqrt{2\kappa^{2}-2\gamma^{2}}}{\kappa},1\right) \\ X_{1+}=\left(\frac{-\sqrt{2}\gamma i+\sqrt{2\kappa^{2}-2\gamma^{2}}}{\kappa},1\right) \end{matrix}$, (C14)

respectively. Therefore, a reduced field amplitude in the transmitting coil for the eigenstate $X_{10}$ in the strongly coupling region ($\kappa>\gamma$), which indicates transmitting coil is little affected by the external environment to achieve better electromagnetic compatibility. Take the non-Hermitian WPT system with $R/r=1.76$ and load impedance of 50 Ω for example ($\kappa/\omega_{0}=0.17$ and $\gamma/\omega_{0}=0.09$), the corresponding normalized intensity distributions for all eigenstates in conventional second-order non-Hermitian resonance and anti-resonance WPT systems are shown in Figs. 10(a) and 10(b), respectively. Compared with Figs. 10(a) and 10(b), it can be found that a dark-mode behavior in the transmitting coil exists in the anti-resonance WPT system at the pinning level ($\omega_{0}$). Moreover, in order to intuitively display the better electromagnetic compatibility of the pinning level in the anti-resonance WPT system, measured magnetic field distributions of RTC and ATC with $R/r=1.76$ in the working state of WPT are shown in Figs. 10(c) and 10(d), respectively. The extremely low field amplitude in Fig. S10(d) indicates the good electromagnetic compatibility of the pinning level in the anti-*PT*-symmetric WPT. Therefore, our results not only provide a new paradigm for the study of anti-*PT*-symmetric non-Hermitian physics from the perspective of synthesis and anti-resonance mode, but also have potential applications in near-field-based stable power transfer with high transmission efficiency for large radius ratio $R/r$, low standby power loss and better electromagnetic compatibility.


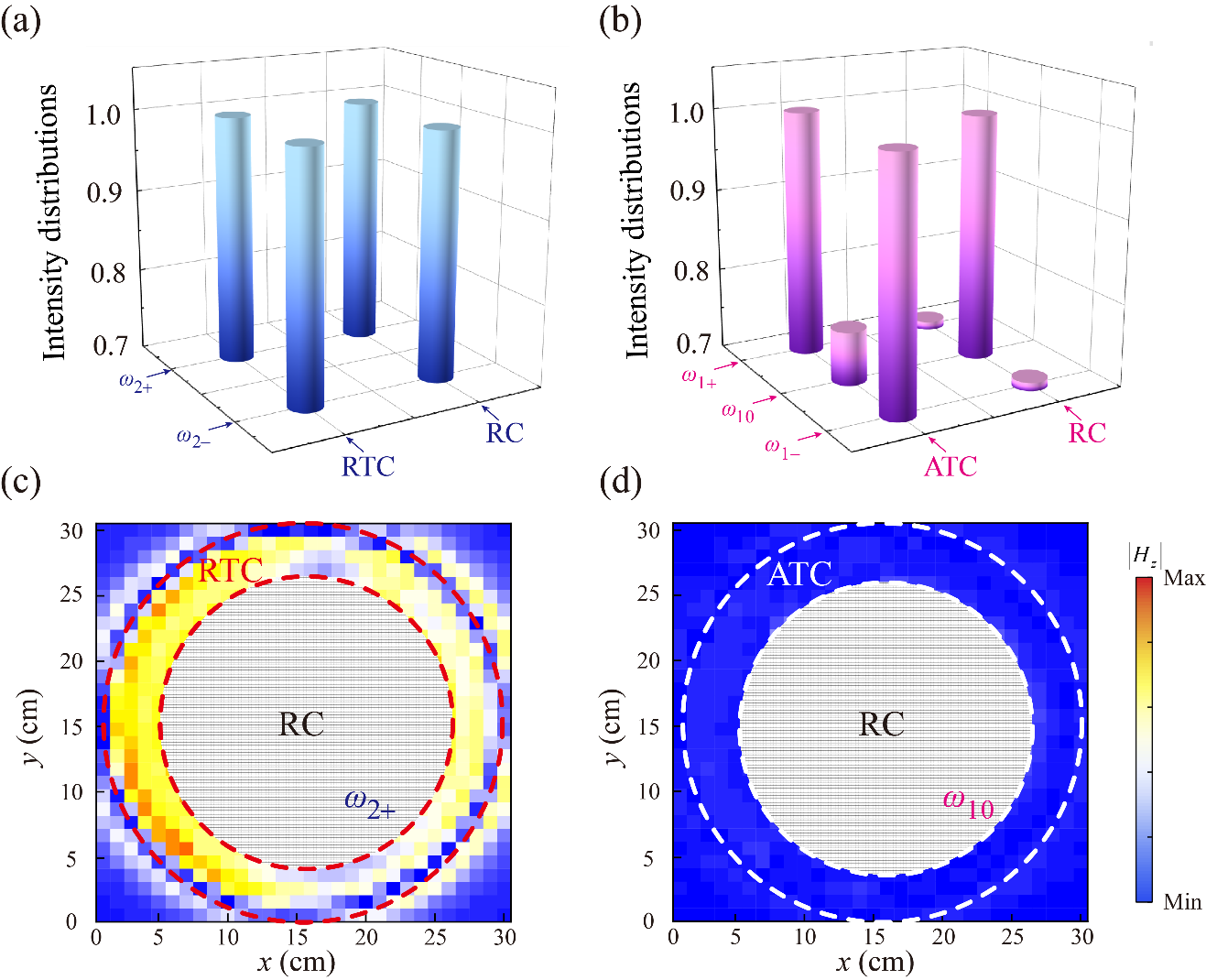


FIG. S10. **Electromagnetic compatibility in the anti-*PT*-symmetric non-Hermitian system.** Intensity distributions for the resonance (a) and anti-resonance (b) WPT systems at different working frequencies. Measured magnetic field distributions of RTC (c) and ATC (d) with $R/r=1.76$ in the working state of the WPT system. The center shaded area indicates the position of the RRC.

## (3) Robustness of level pinning to loads with different power

In practice, in addition to the loads with different sizes considered in the main text, we expect the WPT devices to be valid for diversified loads with different power. For the synthetic anti-*PT*-symmetric WPT system shown in Fig. 3, the evolution of the real and imaginary parts of eigenfrequencies are shown in Figs. S11(a) and S11(b), respectively. Evolution of the calculated (measured) real parts of eigenfrequencies in the synthetic third-order anti-*PT*-symmetric non-Hermitian system as a function of the impedance of loads is marked by the pink solid line (solid spheres) in Fig. S11(a). For comparison, the calculated (measured) phase diagram of conventional WPT with two coils (second-order *PT*-symmetric system) is also given by the blue dashed line (hollow circles). The EPs of the conventional WPT system with RTC and the novel ATC are marked by the ${EP}_{RTC}$ and ${EP}_{ATC}$, respectively. The corresponding evolution of the imaginary parts of eigen-frequencies for two WPT systems is shown in Fig. S11(b). it can be found that the novel pinning level in the anti-*PT*-symmetric system is also effective for loads with different power, because the powers of the loads are proportional to the impendence ($P\sim R$).


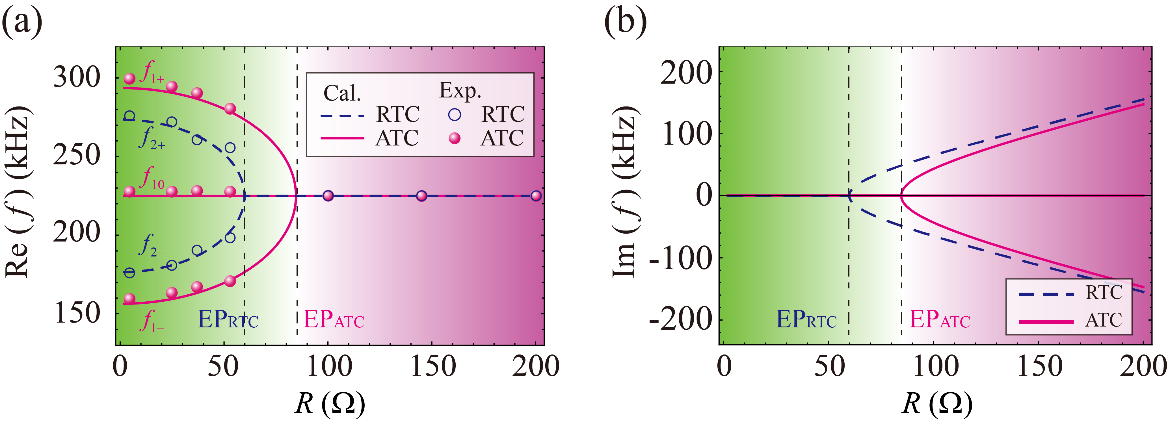


FIG. S11. **Phase diagrams of the synthetic third-order anti-*PT*-symmetric non-Hermitian system when the loads with different impedance**. Evolution of the real (a) and imaginary parts (b) of eigenfrequencies in the synthetic third-order anti-*PT* symmetric and conventional *PT* symmetric non-Hermitian system as a function of the impedance of loads.

## (4) Level pinning to efficient energy transfer with multi-loads

In the main text, the efficient WPT of the synthetic anti-*PT*-symmetric non-Hermitian system has been demonstrated under the condition of large radius ratio of transmitting and receiving coils. In WPT scenarios with small-sized loads, a natural advantage is that the function of multi-loads can be realized. In this section, based on the level pinning in the synthetic anti-*PT-*symmetric non-Hermitian system, efficiency WPT with multi-loads has been investigated. By properly tuning the spacing of the Litz wires, approximately uniform magnetic field distribution in the center of the structure can be achieved. The planar spiral transmission coil designed in the experiment is shown in Fig. S12(a). At the resonance frequency (*f* = 225 kHz), the corresponding measured normalized magnetic field distribution at the center of the transmitting coil is shown in Fig. S12(b). It can be clearly seen that the approximately uniform magnetic field distribution is realized for the designed transmitting coil.


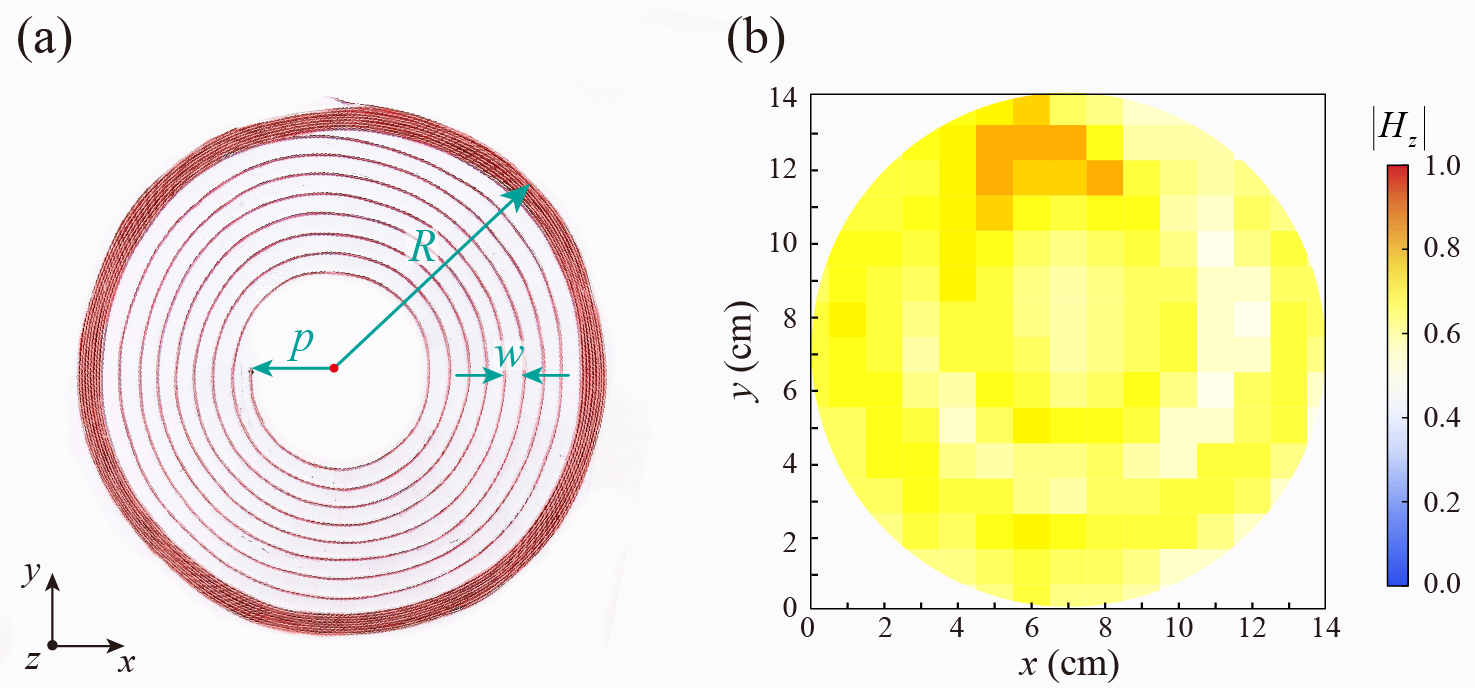


FIG. S12. **Magnetic field distribution of the single transmitting coil.** (a). Photograph of the distributed inductance of the transmitting coil. Here, the corresponding parameters are *R* = 30 cm, *p* = 9 cm, and *w* = 2.3 cm, respectively. (b) Measured magnetic field distributions of transmitting coil at *f* = 225 kHz.

Then, based on the planer transmitting coil, we uncover that the uniform magnetic field distribution can be used to realize the robust WPT when the position of the RRC changed. Considering the RRC moves in the *x* direction with distance $d_{x}$, the corresponding schematic is shown in Fig. S13(a). For the spiral transmitter coil, the magnetic field is nearly uniformed [S2]. Therefore, the coupling strength of the large transmitter coil and the small RRC is always kept nearly a constant, which leads to the transmission efficiency independent of the position of the RRC. Figure S13(b) shows the transmission efficiency of the anti-resonance system in comparison with the conventional resonance WPT system when moving the RRC in the *x* direction from 0 cm to 8 cm when the radius ratio is $R/r=$6, which corresponds to the coupling strength $\kappa_{0}=5.1$ kHz. The calculated and measured transfer efficiencies of the anti-resonance (resonance) system are shown by the blue solid line (pink dashed line) and blue stars (pink circles), respectively. It can be seen that the low transfer efficiency of the conventional resonance WPT system with large radius ratio can be greatly enhanced in the anti-resonance system.

Second, for the multi-loads WPT with *n* independent same loads where the coupling between different loads is ignored, the Kirchhoff’s equations can be expressed as

$\begin{matrix} (i\omega L+1/i\omega C_{1}+1/i\omega C_{0}-Z)I_{1}+I_{2}/i\omega C_{0}=0 \\ I_{1}/i\omega C_{0}+(i\omega L+1/i\omega C_{1}+1/i\omega C_{0})I_{2}+\sum_{j=1}^{n} i\omega M_{j}I_{Aj}=0 \\ i\omega M_{j}I_{2}+(i\omega L+1/i\omega C+Z_{Aj})I_{Aj}=0 (j=1, 2,...,n) \end{matrix}$, (C15)

where $I_{Aj} (j=1, 2,...,n)$ denotes the current in load $Aj$. Similar to Eq. (C3), the dynamic equation of the multi-loads WPT system can be expressed as

$\begin{matrix} \frac{da_{1}}{dt}=(-i\omega_{0}-Z/2L)a_{1}-i(1/2\omega C_{0}L)a_{2} \\ \frac{da_{2}}{dt}=-i\omega_{0}a_{2}-i(1/2\omega C_{0}L)a_{1}+\sum_{j=1}^{n} i(M_{j}\omega/2L)a_{Aj} \\ \frac{da_{Aj}}{dt}=(-i\omega_{0}+Z_{Aj}/2L)a_{Aj}+i(M_{j}\omega/2L)a_{2} (j=1, 2,...,n) \end{matrix}$. (C16)

We first take two loads $A1$ and $A2$ for example to study the multi-loads WPT. The transfer efficiency of the non-Hermitian multi-loads WPT system with ATC can be obtained. Specifically, the transfer efficiencies of the loads at the working frequency are

$\begin{matrix} \eta_{A1}=(2\sqrt{\gamma_{A1}\gamma}\kappa_{0}\kappa_{A1}(-i\gamma_{A2})/\chi)^{2} \\ \eta_{A2}=(2\sqrt{\gamma_{A2}\gamma}\kappa_{0}\kappa_{A2}(-i\gamma_{A1})/\chi)^{2} \end{matrix}$, (C17)

where $\chi$ denotes a constant at the mentioned multi-loads system, $\kappa_{0}=1/2\omega C_{0}L$, $\gamma_{0}=-Z/2L$, $\gamma_{Aj}=-Z_{Aj}/2L$ and $\kappa_{Aj}=-M_{j}\omega/2L$ ($j=1,2$). In the experiment, we first take two same loads $A1$ and $A2$ ($Z_{A1}=Z_{A2}=50 \Omega$ with $\kappa_{0}=5.1$) for example to study the multi-loads WPT, the corresponding schematic is shown in Fig. S13(c). Transfer efficiencies of two RRCs are obtained by randomly moving the positions of $A1$ and $A2$ 15 times. The transfer efficiencies of two same loads are equally divided as $\eta_{A1}=\eta_{A2}=0.21$, as shown in Fig. S13(d). The calculated transfer efficiency is represented by the dashed line, and the measured transfer efficiencies of $A1$ and $A2$ are marked by pink circles and green diamonds, respectively. At the last of this section, two different loads $A_{1}=A$ and $A2=B$ ($Z_{A}\neq Z_{B}$) are introduced to study the multi-loads WPT, the corresponding schematic is shown in Fig. S13(e). The efficiency ratio between two loads is

$\eta_{A}/\eta_{B}=\gamma_{B}\kappa_{A}^{2}/\gamma_{A}\kappa_{B}^{2}$, (C18)

where $\gamma_{A}=-Z_{A}/2L$ ($\gamma_{B}=-Z_{B}/2L$) and $\kappa_{A}=\kappa_{0}$ ($\kappa_{B}=\kappa_{0}^{'}$) denote the radiative loss and coupling strength of the loads $A$ ($B$), respectively. Especially, we take two different load $B$ with $Z_{B}=10 \Omega$ ($\kappa_{0}^{'}=2.1$kHz) and $Z_{B}=100 \Omega$ ($\kappa_{0}^{'}=3.2$kHz) for example. The corresponding transfer efficiencies of case I ($Z_{A}=50 \Omega$, $Z_{B}=10 \Omega$) and case II ($Z_{A}=50 \Omega$, $Z_{B}=100 \Omega$) are shown in Figs. S13(f) and S13(g), respectively. The efficiency ratio for the case I (case II) is near 1.22 (4.99). Therefore, the power redistribution of multi-loads can be flexibly tuned according to the different power of the loads in the anti-*PT*-symmetric WPT system.


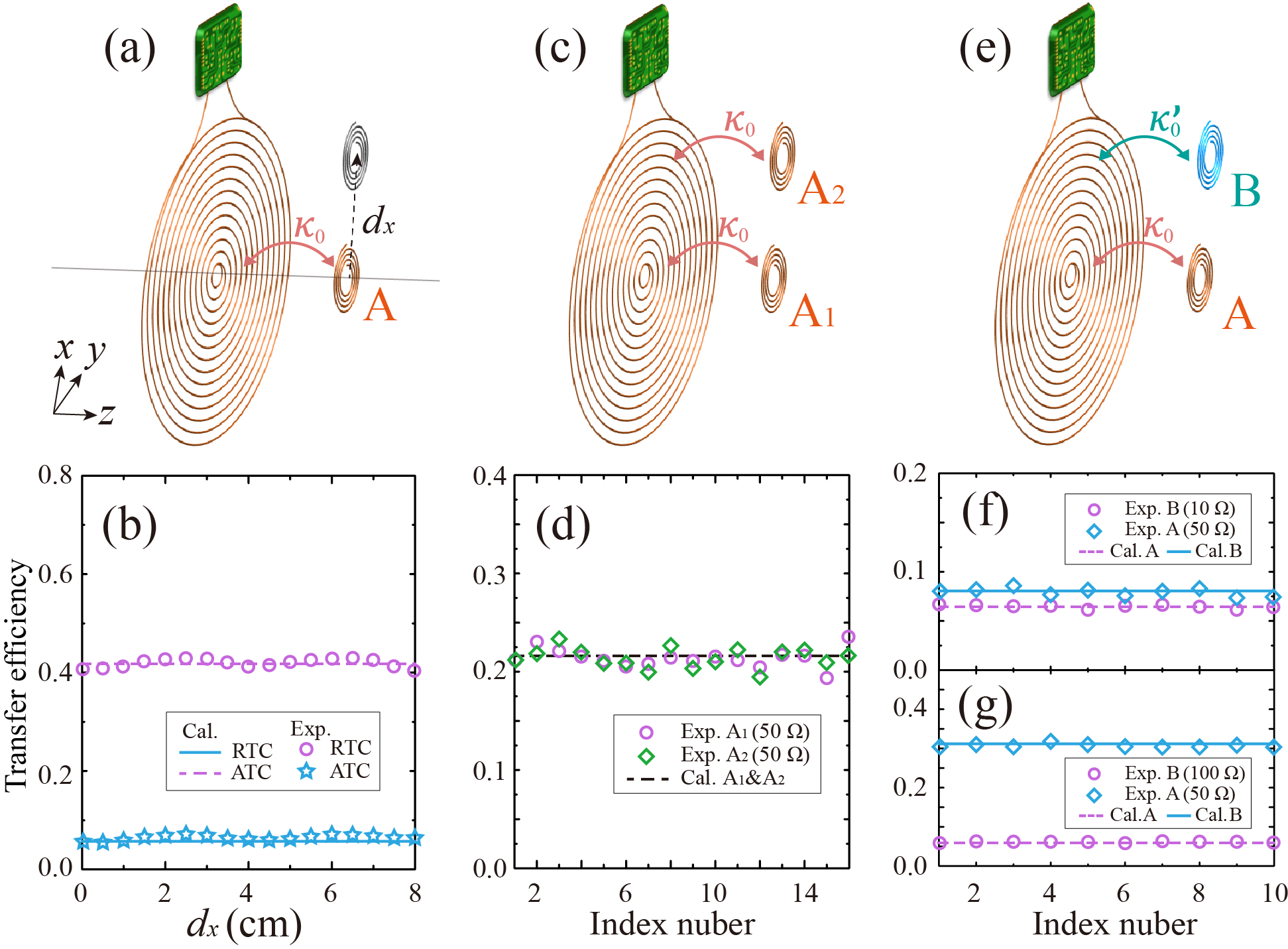


FIG. S13. **Multi-loads WPT in the anti-resonance system.** (a) Schematic of the WPT using ATC when the RRC moves $d_{x}$ in the *x* direction for the radius of the RRC is $r=2.5$ cm. The resistance of RRCs is $Z_{A}=50 \Omega$. (b) The calculated and measured transfer efficiencies of the resonance system (anti-resonance system) by changing the $d_{z}$ from 0 cm to 8 cm, which are shown by the blue solid line (pink dashed line) and blue stars (pink circles), respectively. (c) Schematic of the multi-loads WPT using ATC when RRCs (marked by $A_{1}$ and $A_{2}$) are introduced. The resistance of both RRCs is $Z_{A1}=Z_{A2}=50 \Omega$. (d) Transfer efficiencies of two RRCs are obtained by randomly moving the positions of $A_{1}$ and $A_{2}$ 15 times. The measured transfer efficiencies of $A_{1}$ and $A_{2}$ are marked by pink circles and green diamonds, respectively. (e)-(g) Similar to (c) and (d), but for the RRCs (marked by $A$ and $B$) with different resistance $Z_{A}\neq Z_{B}$. The transfer efficiencies of case I ($Z_{A}=50 \Omega, Z_{B}=10 \Omega$) and case II ($Z_{A}=50 \Omega, Z_{B}=100 \Omega$) are shown in (f) and (g), respectively.

## (5) Experimental setup and details of measurement

For the experimental process, we implement the anti-resonance structure by using the radiofrequency circuit. This synthetic ATC looks like a single coil spatially, thus the anti-*PT*-symmetric WPT system established by the third-order non-Hermitian system composed of ATC and RRC is compact. In the experiment, the toroidal FeSiAl inductor (S106125, 27 mm) is used as the lumped inductor, while the distributed inductor with inductance is fabricated as a coil using Litz wires with 0.078 mm × 400 strands and attaching tightly to the polymethyl methacrylate (PMMA) substrate. In addition, the lumped-metalized polyester film capacitors (the withstand voltage more than 1500 V) are used as the electronic components with capacitance, which are tuned to resonant angular frequency $\omega_{0}=1/\sqrt{LC}$. We measure relevant electrical parameters by using the precision LCR digital bridge (AT2818, Applient). To measure reflection or transmission spectrum, the source and resistance in the circuit diagram are connected to Port 1 and Port 2 of the vector network analyzer in the experiments (Keysight E5071C), respectively. The source impedance is 50 Ω and the power is 0 dBm (1 mW). The entire WPT apparatus can be esteemed as a two-port network (one input port fed by the source and one output port feeding the load). The power transfer efficiency can be expressed as $\eta=\left| S_{21} \right|^{2}$, where $S_{21}$ is the measured linear magnitude transmission coefficient. The inductance of the transmitter and receiver coils is the same as 98 $\mu H$. In the experiment of magnetic field distribution detection, the sample is put on a 10-cm-thick foam substrate ($\varepsilon\sim1$), and then placed on an automatic translation device with scanning steps of 1 cm, which supplies a near-field scanning measurement to probe the field distribution flexibly and accurately. A magnetic probe (small loop with a radius of 2 cm) connecting with the analyzer (in Port 2) is vertically prepared 2 mm above the samples to detect the signals of out-of-plane magnetic field $H_{z}$. By processing the probe signals, we attain the amplitude distributions of the magnetic field $H_{z}$.

## (6) Efficient transfer of WPT with ATC for actual power signal source (PSS)

In fact, the anti-*PT*-symmetric WPT system with ATC can be fully suitable for actual PSS. To verify consistency, according to the suggestions of the reviewer, we use an actual PSS (AG 1006, source impedance 50 Ω, power 10 W) to conduct experimental measurements on the key parameter of robust transfer efficiency. Here, unlike a vector network analyzer (VNA) that can directly obtain transfer efficiency from the measured transmission coefficient S_21_, we need to cooperate with a differential voltage probe (DVP, ETA5010) and a digital oscilloscope (Keysight DSOS054A) to obtain transfer efficiency when using an actual PSS. The photographs of the PPS and DVP are shown in Figs. S14 (a) and S14(b), respectively. Especially, the sinusoidal signal of the PPS is given in Fig. S14(c). Especially, we demonstrate that even if the matching condition is deviated ($\Delta\neq\sqrt{2}\kappa$), the anti-*PT*-symmetric WPT system still supports a mode with slight dissipation at the resonance frequency $\omega=\omega_{0}$, thus its efficient transfer efficiency is more robust than the previous WPT schemes with *PT*-symmetry. The comparison between the VNA and PSS is shown in Fig. S15. For the fixed $\Delta=68.7 kHz$, the NVA and PSS measured transfer efficiency of the anti-*PT*-symmetric WPT systems with ATC as a function of $R/r$ are shown by pink and red circles. Moreover, considering the optimized working frequency $\omega_{-}$=198.3 kHz for the case of $r=$8.5 cm in the *PT*-symmetric system with RTC, the NVA and PSS measured transfer efficiency at the fixed frequency as a function of $R/r$ are shown by cyan and blue stars, respectively. From Figs. 4(c) and S15, it can be clearly seen that whether the anti-*PT*-symmetric system meets the optimization conditions or not, it also has better transfer efficiency compared to traditional linear *PT*-symmetric WPT systems. Especially, for a clearer comparison, tables S2 and S3 show the S_21_ and voltage measured by VNA and PSS, respectively. The NVA and PSS measured results meet well with each other. Therefore, the efficient energy transfer results achieved using the *PT*-symmetry obtained by the NVA can be extended to actual PSS. In addition, it should be noted that in order to accurately measure the magnetic field distributions, we have used an automatic translation device that can match the VNA. Using actual PSS and DPV cannot be compatible with the automatic translation device, and testing may bring additional errors. Therefore, we request the reviewer to allow us to retain the results of the VNA to clearly show the new physical principles of anti-*PT*-symmetry to achieve a new WPT scheme.


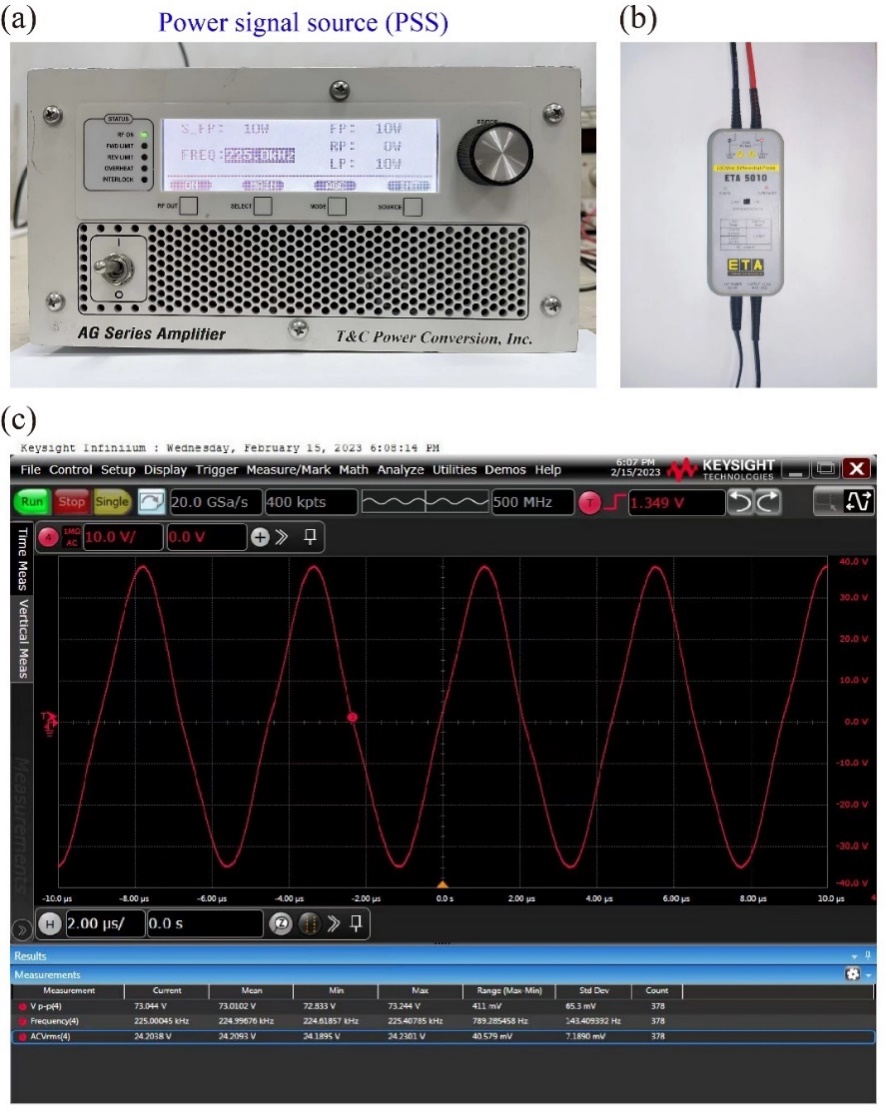


FIG. S14. **The actual PSS for the measurement of transfer efficiency**. (a) Photograph of the actual power signal source (PSS, AG 1006). (b) Photograph of the differential voltage probe (DVP, ETA5010). (c) The sinusoidal signal of the PPS given by a digital oscilloscope (Keysight DSOS054A).


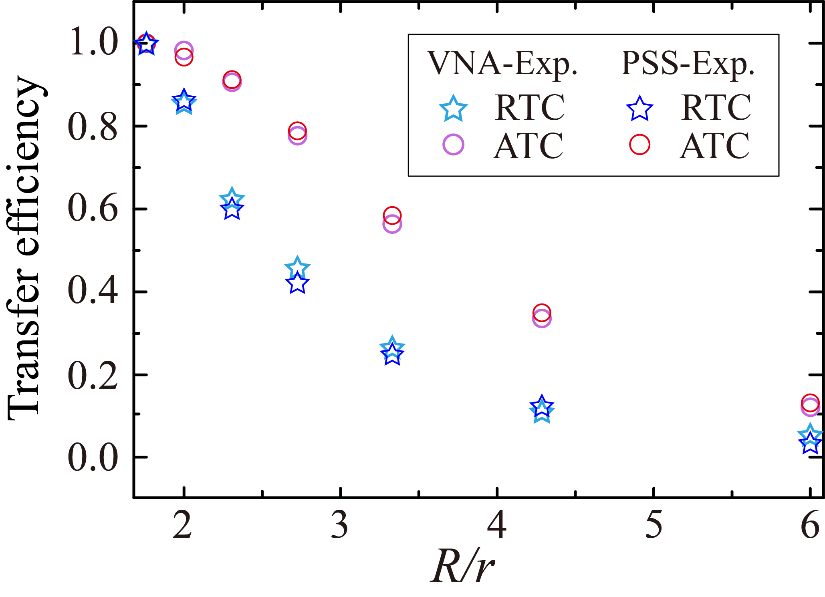


FIG. S15. **Transfer efficiency of the two kinds of WPT systems as a function of** $\boldsymbol{R/r}$. The NVA and PSS measured results of the WPT system with RTC (ATC) at $\omega_{-}$=198.3 kHz ($\omega_{0}$=225 kHz) are shown by the cyan stars (pink circles) and cyan stars (red circles), respectively.

| *r* | 4.5 cm | 5.5 cm | 6.5 cm | 7.5 cm | 8.5 cm |
| --- | --- | --- | --- | --- | --- |
| S_21_ | 0.504 | 0.658 | 0.767 | 0.902 | 0.975 |
| $\eta_{s21}$ | 0.254 | 0.433 | 0.588 | 0.814 | 0.95 |
| U (V) | 10.88 | 14.17 | 16.89 | 20.31 | 21.80 |
| $\eta_{U}$ | 0.237 | 0.401 | 0.571 | 0.824 | 0.95 |

**Table S2.** Transfer efficiency of the WPT system with RTC.

| *r* | 4.5 cm | 5.5 cm | 6.5 cm | 7.5 cm | 8.5 cm |
| --- | --- | --- | --- | --- | --- |
| S_21_ | 0.731 | 0.860 | 0.927 | 0.965 | 0.973 |
| $\eta_{s21}$ | 0.535 | 0.740 | 0.860 | 0.932 | 0.95 |
| U (V) | 16.63 | 19.33 | 20.80 | 21.43 | 21.79 |
| $\eta_{U}$ | 0.553 | 0.747 | 0.866 | 0.919 | 0.95 |

**Table S3.** Transfer efficiency of the WPT system with ATC.

By using the PSS, we further demonstrate intuitively the efficient power transfer scheme by replacing the receiver’s load with a light-emitting diode (LED) lamp connected to a non-resonant coil in the weak coupling region (*R/r*=4.3). In this case, the transmission efficiency of ATC is approximately four times that of RTC. From Fig. S16, it can be seen that the LED lamp is illuminated in the system with ATC while remaining dark in the system with RTC.


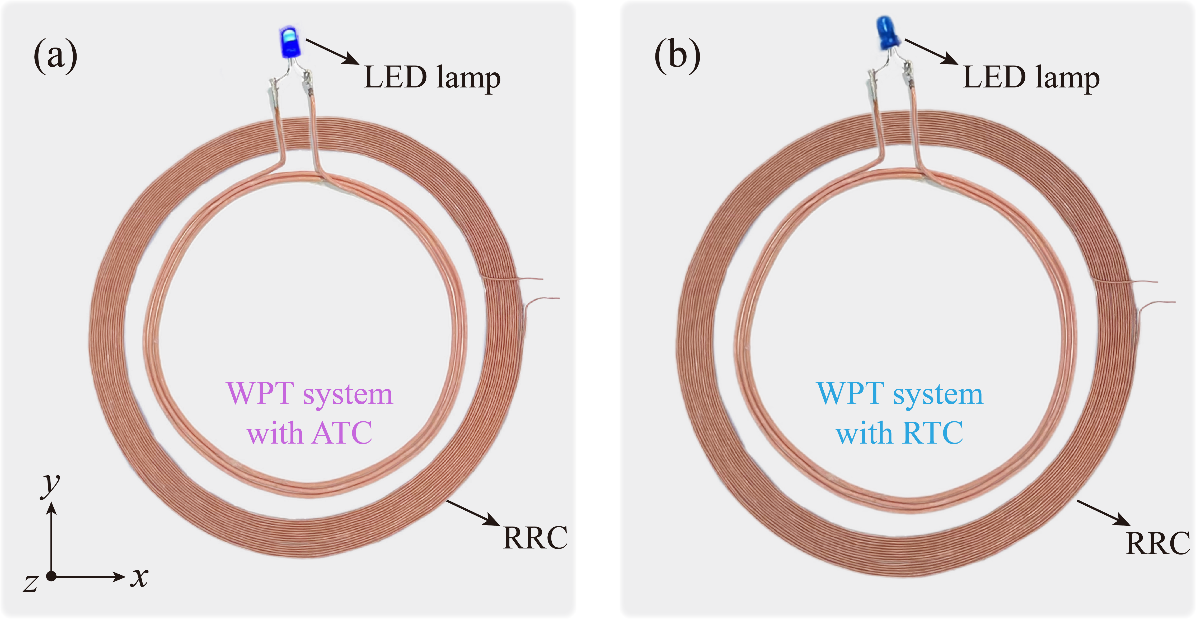


FIG. S16. **Experimental demonstration of the efficient power transfer for lighting the LED lamp**. (a) The WPT system with ATC. (b) Same as (a), but for the case with RTC.

## (7) Extended to higher order circuit model

In the main text, based on the near-field coupling between the anti-resonance mode and resonance mode, we have studied the second-order non-Hermitian system with third-order anti-*PT*-symmetry. In this part, we will introduce the synthetic method for high-order anti-*PT*-symmetric WPT system can be extended to higher order models. Take the fifth order anti-*PT*-symmetric WPT system for example, as shown in Fig. S17. In this Fifth-order anti-*PT*-symmetric WPT system with two synthetic circuits, the Kirchhoff’s equations can be expressed as

$\begin{matrix} (i\omega L_{1}+1/i\omega C_{1}+1/i\omega C_{0}+Z)I_{1}+I_{2}/i\omega C_{0}=0 \\ I_{1}/i\omega C_{0}+(i\omega L_{2}+1/i\omega C_{2}+1/i\omega C_{0})I_{2}+i\omega MI_{3}=0 \\ i\omega MI_{2}+(i\omega L_{3}+1/i\omega C_{5}+1/i\omega C_{3})I_{3}+I_{4}/i\omega C_{3}=0 \\ I_{3}/i\omega C_{3}+(i\omega L_{4}+1/i\omega C_{4}+1/i\omega C_{6})I_{4}+I_{5}/i\omega C_{4}=0 \\ (i\omega L_{5}+1/i\omega C_{4}+1/i\omega C_{7}+R)I_{5}+I_{4}/i\omega C_{4}=0 \end{matrix}$. (C19)

where $M$ denotes the mutual inductance between the synthetic ATC and synthetic RRC. Here, in order to simplify the system, we consider that $C_{1}=C_{2}=C_{5}=C_{6}=C_{7}=C_{I}$, $C_{0}=C_{3}=C_{4}=C_{II}$, $L_{1}=L_{2}=L_{3}=L_{4}=L_{5}=L$, and $Z=-R$. Similar to Eq. (C1), the equations Eq. (C19) can be rewritten as

$\begin{matrix} (i\omega^{2}+1/iLC_{1}+1/iLC_{0}-R\omega/L)a_{1}+a_{2}/iLC_{0}=0 \\ a_{1}/iLC_{0}+(i\omega^{2}+1/iC_{2}L+1/iC_{0}L)a_{2}+(i\omega^{2}M/L)a_{3}=0 \\ i\omega^{2}M/La_{2}+(i\omega^{2}+1/iC_{5}L+1/iC_{3}L)a_{3}+a_{4}/iC_{3}L=0 \\ a_{3}/iC_{3}L+(i\omega^{2}+1/iC_{4}L+1/iC_{6}L)a_{4}+a_{5}/iC_{4}L=0 \\ (i\omega^{2}+1/iC_{4}L+1/iC_{7}L+\omega R/L)a_{5}+a_{4}/iC_{4}L=0 \end{matrix}$. (C20)

After we define $1/C=1/C_{I}+1/C_{II}$, $\omega_{0}=1/\sqrt{LC}$, and make an appropriate approximation $\omega^{2}-\omega_{0}^{2}\approx2\omega(\omega-\omega_{0})$, we can obtain the dynamic equation derived from the Kirchhoff’s equations as

$\begin{matrix} \frac{da_{1}}{dt}=-i\omega_{0}a_{1}-R/2La_{1}-i\frac{\omega_{0}}{2}\frac{C}{C_{II}}a_{2} \\ \frac{da_{2}}{dt}=-i\omega_{0}a_{2}-i\frac{\omega_{0}}{2}\frac{C}{C_{II}}a_{1}+i\frac{M}{L}\frac{\omega_{0}}{2}a_{3} \\ \frac{da_{3}}{dt}=-i\omega_{0}a_{3}+i\frac{M}{L}\frac{\omega_{0}}{2}a_{2}-i\frac{\omega_{0}}{2}\frac{C}{C_{II}}a_{4} \\ \frac{da_{4}}{dt}=-i\omega_{0}a_{4}-i\frac{\omega_{0}}{2}\frac{C}{C_{II}}a_{3}-i\frac{\omega_{0}}{2}\frac{C}{C_{II}}a_{5} \\ \frac{da_{5}}{dt}=-i\omega_{0}a_{5}+R/2La_{5}-i\frac{\omega_{0}}{2}\frac{C}{C_{II}}a_{4} \end{matrix}$. (C21)

Moreover, we define $\gamma_{0}=R/2L$, $\kappa=\omega_{0}C/2C_{II}$, and use a suitable unitary transformation $a_{1}=(a_{+2}+a_{-2})/\sqrt{2}$, $a_{2}=(a_{+2}-a_{-2})/\sqrt{2}$, $a_{4}=(a_{+1}-a_{-1})/\sqrt{2}$, $a_{5}=(a_{+1}+a_{-1})/\sqrt{2}$,$a_{3}=a_{0}$, the dynamic equation of the system becomes

$H\left(\begin{matrix} a_{+2} \\ a_{+1} \\ a_{0} \\ a_{-1} \\ a_{-2} \end{matrix}\right)=\omega\left(\begin{matrix} a_{+2} \\ a_{+1} \\ a_{0} \\ a_{-1} \\ a_{-2} \end{matrix}\right)$. (C22)

When condition $M=-C_{V}/\omega_{0}^{2}$ is satisfied, the equivalent Hamiltonian of the system can be written as

$H=\left(\begin{matrix} \omega_{0}+\kappa_{1}-i\gamma& 0 & -\kappa& 0 & -i\gamma\\ 0 & \omega_{0}+\kappa_{1}+i\gamma& \kappa& i\gamma& 0 \\ -\kappa& \kappa& \omega_{0} & -\kappa& \kappa\\ 0 & i\gamma& -\kappa& \omega_{0}-\kappa_{1}+i\gamma& 0 \\ -i\gamma& 0 & \kappa& 0 & \omega_{0}-\kappa_{1}-i\gamma\end{matrix}\right)$, (C23)

where $\gamma=\gamma_{0}/2$, and $\kappa=\kappa_{2}/\sqrt{2}$. From Eq. (C23), we can clearly see that the fifth-order anti-*PT*-symmetry of the system about the center $\omega_{0}$ is verified $(PT)H(PT)^{-1}=PH^{*}P=-H$.


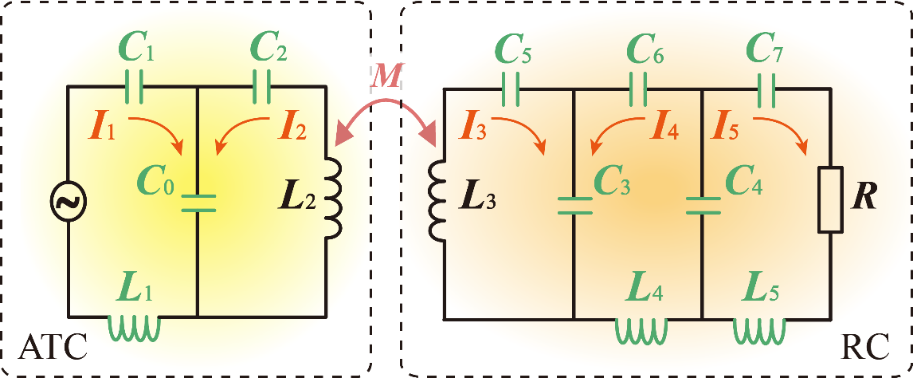


**Fig. S17.** **The effective circuit model of the synthetic fifth-order anti-*PT*-symmetric WPT system**. Fifth-order anti-*PT*-symmetric WPT system with two synthetic circuits, one of which is a second-order circuit and the other is a third-order circuit.

From Eq. (C23), we can obtain the eigenvalues of the system as

$\begin{matrix} \omega_{1}=\omega_{0}, \\ \omega_{2,3}=\omega_{0}\pm\sqrt{A-B}, \\ \omega_{4,5}=\omega_{0}\pm\sqrt{A+B}, \end{matrix}$ (C24)

where $A=-2\gamma^{2}+2\kappa^{2}+\kappa_{1}^{2}$ and $B=2\sqrt{(\gamma^{2}+\kappa^{2})^{2}-\gamma^{2}\kappa_{1}^{2}}$. Here, without the loss of generality, we assume $\omega_{0}=0$, $\gamma=1$, and $\kappa=0.5$, the evolution of real and imaginary parts of eigenfrequencies in the synthetic fifth-order anti-*PT*-symmetric non-Hermitian system when the coupling coefficient $\kappa_{1}$ changes, which are shown by the solid pink line and dashed blue line in Fig. S18, respectively. Especially, two pairs of eigenvalues degenerate, thus there are three modes existed in the eigenvalue spectrum. The high-order non-Hermitian physical system has been proposed to have very unique physical properties, such as realizing high-sensitivity sensing [S3]. Moreover, it should be emphasized that similar to the third-order anti-*PT*-symmetric non-Hermitian system, the pure real eigenmode $\omega=\omega_{0}$ independent of the coupling coefficient can be used for robust and efficient WPT.


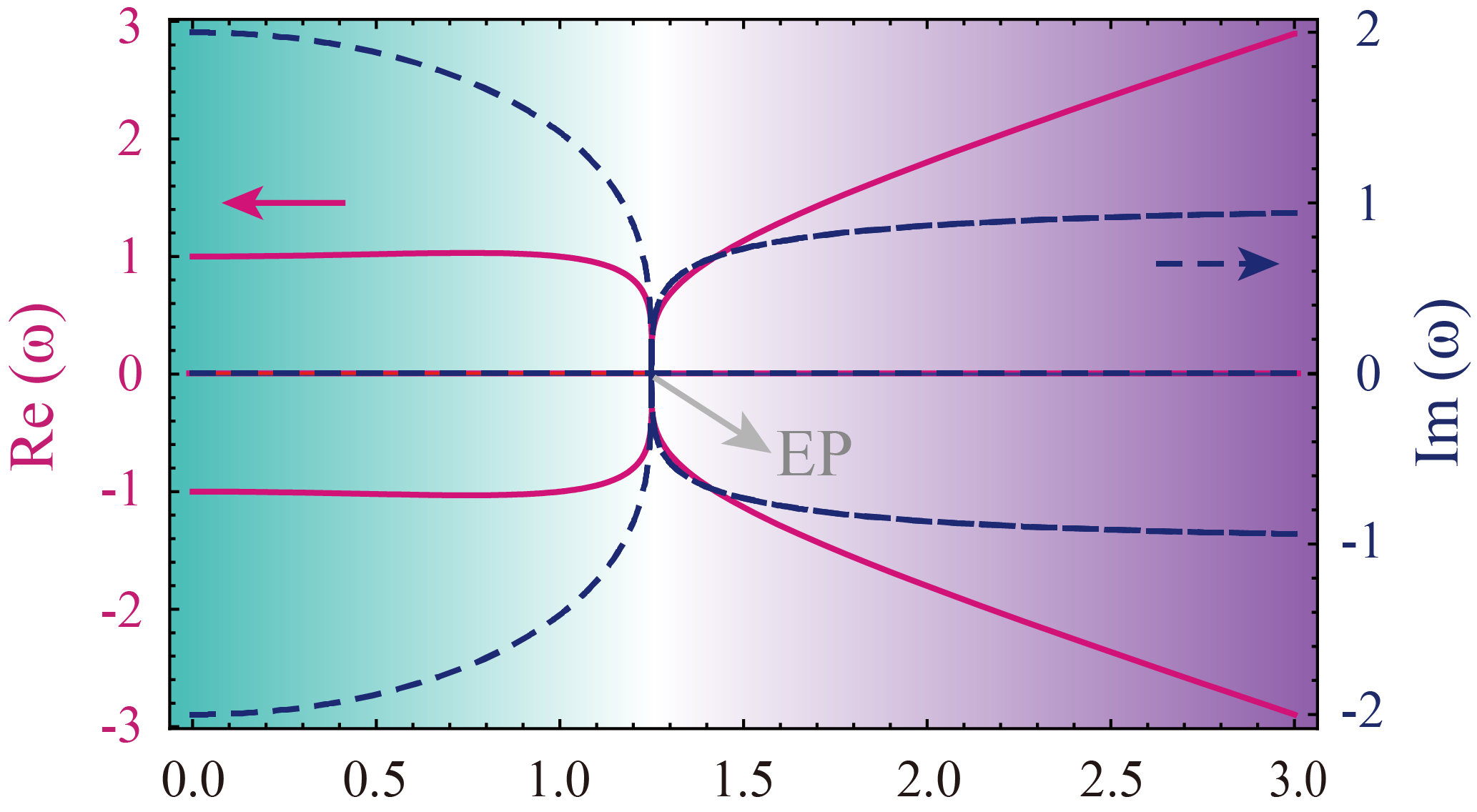


FIG. S18. **Phase diagrams for the synthetic fifth-order anti-*PT*-symmetric non-Hermitian system**. Evolution of the real parts (left) and imaginary part (right) of eigenfrequencies in the synthetic fifth-order anti-*PT*-symmetric non-Hermitian system as a function of the coupling coefficient $\kappa_{1}$.

**Supplementary references**

[S1] Y. Sun, W. Tan, H. Q. Li, J. Li, and H. Chen, Experimental demonstration of a coherent perfect absorber with PT phase transition, *Phys. Rev. Lett.* 112, 143903 (2014).

[S2] J. Song, F. Q. Yang, Z. W. Guo, X. Wu, K. J. Zhu, J. Jiang, Y. Sun, Y. H. Li, H. T. Jiang, and H. Chen, Wireless power transfer via topological modes in dimer chains, *Phys. Rev. Appl*. 15, 014009 (2021).

[S3] W. Chen, S. Kaya Ozdemir, G. Zhao, J. Wiersig and L. Yang, Exceptional points enhance sensing in an optical microcavity, *Nature* 548, 192 (2017).
